# Integrative biology of extracellular vesicles in diabetes mellitus and diabetic complications

**DOI:** 10.7150/thno.65778

**Published:** 2022-01-01

**Authors:** Jing Liu, Yanyan Zhang, Yan Tian, Wei Huang, Nanwei Tong, Xianghui Fu

**Affiliations:** 1 Division of Endocrinology and Metabolism, National Clinical Research Center for Geriatrics, State Key Laboratory of Biotherapy and Cancer Center, West China Medical School, West China Hospital, Sichuan University and Collaborative Innovation Center of Biotherapy, Chengdu, China.; 2 Department of Geriatric Medicine, Lanzhou University Secondary Hospital, Lanzhou, Gansu, China.; 3 Department and Laboratory of Integrated Traditional Chinese and Western Medicine, Sichuan Provincial Pancreatitis Centre and West China-Liverpool Biomedical Research Centre, West China Hospital, Sichuan University, Chengdu, China.; 4 Division of Endocrinology and Metabolism, Center for Diabetes and Metabolism Research, Laboratory of Diabetes and Islet Transplantation Research, West China Medical School, West China Hospital, Sichuan University, Chengdu, China.

**Keywords:** diabetes mellitus, extracellular vesicles, biogenesis, sorting, adipocytes, macrophages, islet, endothelial cells

## Abstract

Diabetes mellitus (DM) is a chronic systemic disease with increasing prevalence globally. An important aspect of diabetic pathogenesis is cellular crosstalk and information exchange between multiple metabolic organs and tissues. In the past decade, increasing evidence suggested that extracellular vesicles (EVs), a class of cell-derived membrane vesicles that transmit information and perform inter-cellular and inter-organ communication, are involved in the pathological changes of insulin resistance (IR), inflammation, and endothelial injury, and implicated in the development of DM and its complications. The biogenesis and cargo sorting machinery dysregulation of EVs may mediate their pathogenic roles under diabetic conditions. Moreover, the biogenesis of EVs, their ubiquitous production by different cells, their function as mediators of inter-organ communication, and their biological features in body fluids have generated great promise as biomarkers and clinical treatments. In this review, we summarize the components of EV generation and sorting machinery and highlight their role in the pathogenesis of DM and associated complications. Furthermore, we discuss the emerging clinical implications of EVs as potential biomarkers and therapeutic strategies for DM and diabetic complications. A better understanding of EVs will deepen our knowledge of the pathophysiology of DM and its complications and offer attractive approaches to improve the prevention, diagnosis, treatment, and prognosis of these disorders.

## Introduction

Diabetes mellitus (DM), a systemic disease with an alarming increase in incidence worldwide, is characterized by chronic hyperglycemia resulting from insulin resistance (IR) and/or insulin secretion deficiency. The International Diabetes Federation estimates that 9.3% of adults aged 20-79 years are currently living with DM and this prevalence is projected to rise to 10.2% by 2030 and to 10.9% by 2045 1. DM and associated complications account for 11.3% of global deaths from all causes 1. Moreover, DM is an independent risk factor for various diseases, including coronary heart disease, stroke, cancer, chronic kidney disease, blindness, and lower limb amputation, placing a heavy burden on global health 2-6. There are no efficacious pharmacological treatments for DM, primarily due to its complicated pathophysiologic mechanisms and unclear etiopathogenesis.

Generally, DM can be mainly divided into type 1 diabetes (T1D) and type 2 diabetes (T2D). T2D is the most common type of DM, accounting for about 90% of cases worldwide, whereas T1D constitutes more than 10% 7, 8. Although the etiologies of T1D and T2D are distinct, the progression of both diseases is primarily due to dysregulated intercellular and inter-organ communication. In T1D, the interaction between immune cells and pancreatic islets contributes to the dysfunction and death of β cells. More recently, the involvement of gut microbiota in the auto-immune response against β cells has added another layer of complexity to T1D pathogenesis 9. In T2D, intricate inter-organ communication among the pancreas, adipose tissue (AT), liver, muscle, intestine, hypothalamus, and other tissues plays an essential role in hyperglycemia and IR, two hallmarks of T2D 10. For example, increased inflammatory cytokines and free fatty acids (FFAs) derived from obese AT can induce lipotoxicity and IR in the liver and skeletal muscle, and also impair the glucoregulatory function of the central nervous system (CNS) and gut, in turn, perturbing the AT secretome and reinforce its IR 11. Furthermore, systemic IR triggers a rise in insulin demand, overstressing β cells and eventually resulting in islet dysfunction and relative insulin insufficiency. Therefore, dysregulated inter-organ communication plays a key role in initiating and amplifying the deleterious vicious cycle of IR and hyperglycemia in T2D. Under these circumstances, signaling molecules mediating inter-organ conversation are likely key pathogenic factors for T1D and T2D. Indeed, several classes of signaling mediators, including adipokines, hepatokines, peptides from CNS, and hormones from the pancreas and intestine, are crucial in the initiation and progression of both T1D and T2D 12-18, and therapeutic strategies targeting these molecules have been partially applied in the clinic benefiting patients.

Recently, extracellular vesicles (EVs) have emerged as a novel class of signaling molecules mediating intercellular and inter-organ communication. Released by various cell types, EVs are widely distributed in diverse tissues and body fluids. Moreover, bioactive contents loaded in EVs including proteins, DNAs, RNAs, lipids, and metabolites, are protected by the lipid bilayer membrane against harsh environments and prevented from degradation and digestion. The size, quantity, morphology, cargoes, and other characteristics of EVs are highly variable and influenced by the parental cell type. Because of these fundamental features, EVs are well-suited to serve as versatile carriers and transporters transmitting signals from parental cells to recipient cells. Correspondingly, EVs have been shown to regulate various biological and physiological processes and are implicated in multiple human diseases, such as cancer, cardiovascular diseases, metabolic disorders, and neurodegenerative diseases 19. In particular, understanding the role of EVs in the crosstalk among multiple metabolic tissues would provide a new perspective to understand the pathogenesis of DM and diabetic complications and develop therapeutic strategies.

Here we outline the current knowledge of diabetic pathogenesis, focusing on the potential mechanisms underlying the altered EV biogenesis in DM and the role of EVs originating from different cells in regulating systemic metabolism. Finally, we summarize studies of EV-RNAs as markers and discuss potential applications of EVs derived from native cells to treat DM and diabetic complications.

## Pathology of DM and its complications

IR, also known as low insulin sensitivity, is an essential mechanism underlying T2D occurrence and a critical driver of associated complications 20. Although there is no consensus on the molecular mechanism(s) triggering IR, inter-organ communication has been widely recognized as a key contributor 21. During obesity, massive expansion of AT, often accompanied by inadequate vascularization, induces hypoxic response and inflammation, leading to increased infiltration of pro-inflammatory macrophages and inflammatory cytokine release 22. Inflammation can further disturb insulin signaling in AT, resulting in enhanced lipolysis and increased release of FFAs and adipokines into circulation. Subsequently, elevated circulating FFAs elicit lipotoxicity and impair insulin action in the liver and skeletal muscle 23, 24. Also, downstream pathways aroused by IR cooperatively induce reactive oxygen species (ROS) production and systemic inflammation, further worsening IR. Consequently, IR suppresses plasma membrane translocation of the glucose transporter (GLUT) and glucose uptake, leading to elevated blood glucose levels and systemic energy metabolism disturbance. In addition to classic metabolic tissues, other organs such as the gut, vascular endothelium, and brain, have recently been shown to participate in the development of IR and T2D 25-29. For example, vascular endothelium can function as an adjustable barrier to control the transport of metabolic macromolecules such as FFAs, lipoproteins, and glucose to metabolic organs, including the skeletal muscle and AT. Bioactive molecules secreted by endothelial cells (ECs), for e.g., nitric oxide and growth factors, may modulate systemic metabolism by modulating insulin sensitivity, maintaining pancreatic islet structure, and insulin secretion 30, 31.

Pancreatic β cell failure, another hallmark of T2D, is also associated with inter-organ communication. Increased demand for insulin, typically due to peripheral IR, leads to excess insulin secretion and elevated islet amyloid polypeptide (IAPP) production. Simultaneously expressed and secreted with insulin, IAPP is a membrane-permeant toxic agent, and its accumulation forms amyloid deposits, causing pancreatic damage 32. Meanwhile, chronic elevated FFAs and glucose elicit endoplasmic reticulum (ER) stress and inflammatory response in islets, which aggravate the pancreatic injury and compromise insulin secretion 33, 34. In this circumstance, the combination of IR and β cell decompensation contributes to overt T2D.

Insulin deficiency, primarily resulting from reduced β cell function and mass, is the major driver of T1D. Insulitis induced by autoimmune response results in β cell death and chronic autoantigen exposure, reamplifying the immune attack 35. The uncontrollable autoimmune response against β cells accounts for T1D pathology. Besides the crosstalk between the islet and immune cells, gut and immune system communication has also been implicated in T1D pathogenesis. For example, loss of gut integrity and changes in metabolites caused by enteric dysbacteriosis can significantly promote innate and adaptive autoimmune response against islet cells, thereby participating in T1D development 36-39.

The development of diabetic complications shares multiple pathological processes with DM, such as glucose variability, lipotoxicity, and activation of advanced glycation end products (AGEs) and receptors for AGE (RAGEs) signaling, and consequent mitochondrial dysfunction, oxidative stress, epigenetic changes, and inflammation response 40-47. Besides the in-depth understanding of underlying molecular mechanisms, the significance of inter-organ crosstalk in the pathogenesis of diabetic complications has recently been emphasized. For instance, there is increased lipoprotein secretion by insulin-resistant hepatocytes that can be glycated and oxidated, leading to renal lipid metabolism disorder and promoting the development of diabetic nephropathy (DN) 48-51.

**Figure 1:**
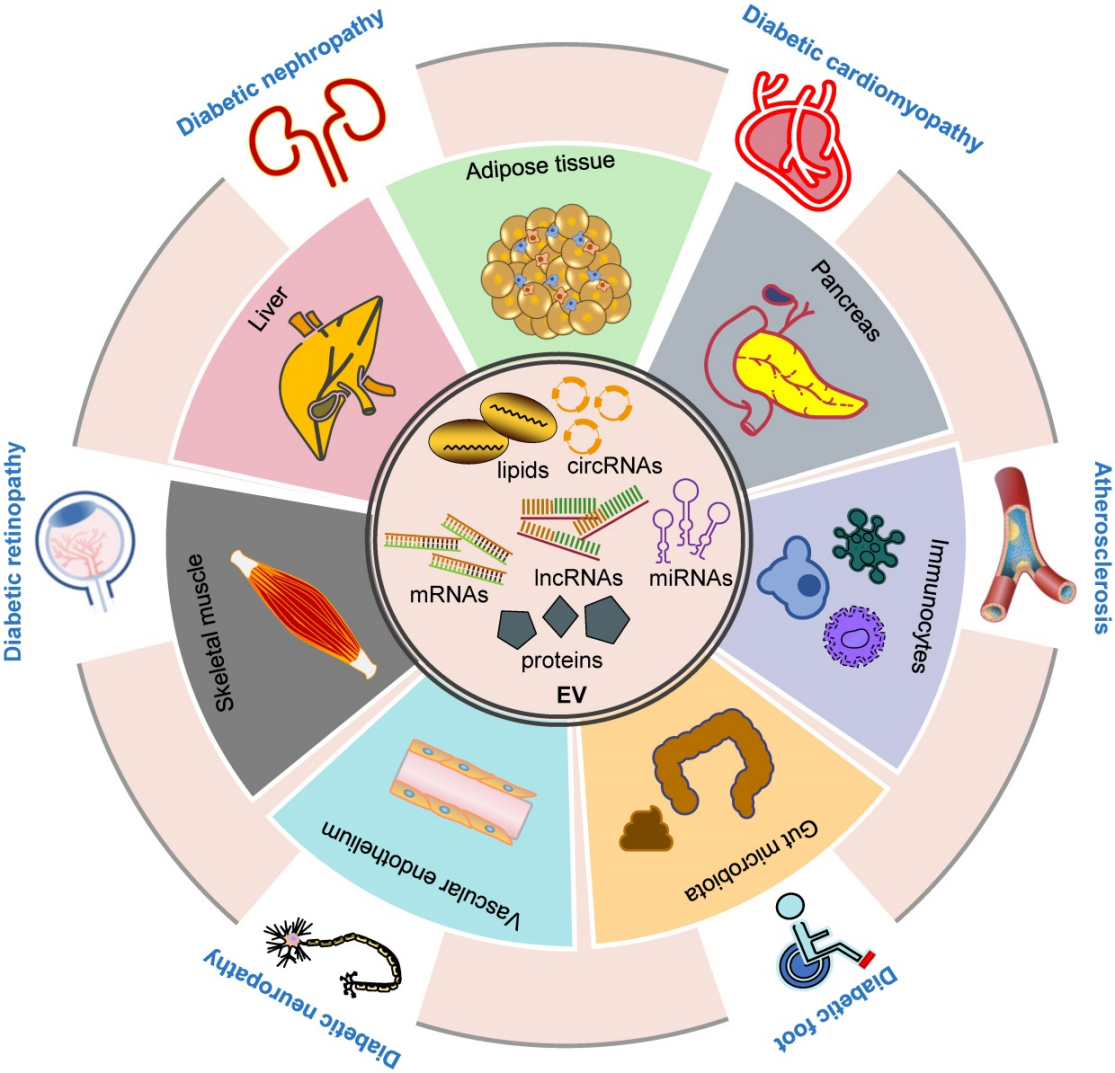
** Inter-organ crosstalk mediated by EVs in the pathogenesis of DM and diabetic complications.** EVs contain different proteins, RNAs, DNAs and lipids (inner circle). EVs participate in the development of DM and its complications via multiple ways. EVs derived from various tissues, including adipose, liver, pancreas, skeletal muscle, immunocytes, vascular endothelium and gut microbiota, play a role in the development and progression of DM (inner ring). Moreover, these EVs are involved in the pathogenesis of diabetic complications including diabetic foot, cardiomyopathy, nephropathy, retinopathy, neuropathy and atherosclerosis (outer ring). **Abbreviations:** circRNAs: circular RNAs; DM: diabetes mellitus; EV: extracellular vesicle; lncRNAs: long noncoding RNAs; miRNAs: microRNAs.

Furthermore, inter-organ and intercellular communication are crucial in the pathogenesis of DM and diabetic complications, and our current understanding can only be considered as the tip of the iceberg. In this context, EVs, an emerging mediator of intra- and inter-organ crosstalk, have been shown to play a critical role in various pathological pathways of DM and its complications (Figure 1), providing a novel paradigm in pathological mechanisms and therapeutic interventions. Future investigation is required to delineate the molecular mechanisms of EV-mediated signaling under diabetic conditions and further explore their implications in treating these disorders.

## EVs

Based on their biogenesis, EVs can be divided into two major groups, exosomes (30-100 nm) and microvesicles (MVs, 50-1000 nm) (also known as ectosomes, microparticles) 52. MVs are generated directly by budding and shedding from the plasma membrane, while exosome generation involves intraluminal vesicle (ILV) budding and shedding, intracellular multivesicular endosome (MVE) trafficking, and ILV release 53, 54. In this section, we mainly introduce EVs from two perspectives: (i) generative processes and (ii) mechanisms of cargo sorting.

### EV generation

**Figure 2:**
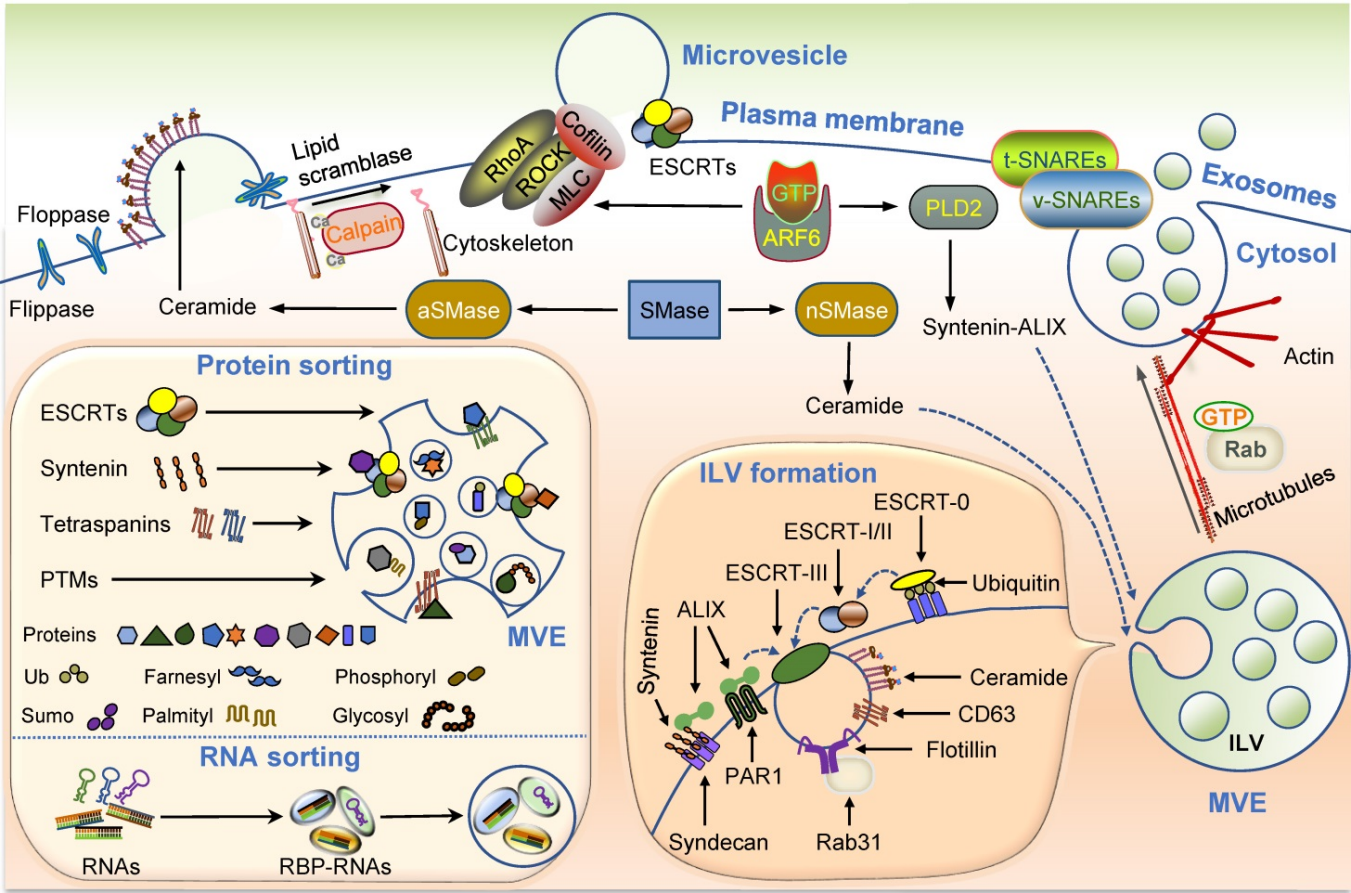
** EV biogenesis and cargo sorting.** Microvesicles and exosomes are two major categories of EVs. Microvesicles are released directly from plasma membrane budding and shedding. Exosomes are generated by inward budding of endosomes, known as MVEs, which fuse to plasma membrane, and are followed by the release of exosomes. Multiple molecules are implicated in the biogenesis of microvesicles and exosomes, such as ESCRT complexes and related proteins, ceramide, SMase, syntenin, syndecan, calpain, Rab GTPases, and so on (see text). Exosomes contain different types of proteins and RNAs, whose sorting are modulated by several molecules, including ESCRT complexes, syntenin, tetraspanins, and RBPs. PTMs on certain proteins also have a role in the sorting of exosomal cargos. **Abbreviations:** aSMase: acid sphingomyelinase; ESCRT: endosomal sorting complex required for transport; EVs: extracellular vesicles; ILV: intraluminal vesicle; MVE: multivesicular endosome; nSMase: neutral sphingomyelinase; PTMs: post-translational modifications; RBP: RNA binding protein; SMase: sphingomyelinase, SNARE: soluble N-ethylmaleimide-sensitive fusion attachment protein receptor; t-SNAREs: target-membrane SNAREs; v-SNAREs: vesicle-membrane SNAREs.

Both exosomes and MVs are vesicles formed by membrane budding away from the cytosol, and their generation requires an integrative cytoskeleton and membrane reorganization (Figure 2).

#### Exosome generation

The generation of exosomes principally consists of biogenesis, transport, and release. ILV formation is the first step of exosome generation, which depends on the endosomal sorting complex required for transport (ESCRT). Several ESCRT-independent ILV formation pathways mediated by ceramide-, CD63-, Rab31, and others have been detected 55-58. As the best known ESCRT-independent mode, cone-shaped lipid ceramide enriched in specific microdomains of the endosomal membrane can effectively lead to membrane curvature alteration and budding of ILVs 55. Tetraspanin CD63, the specific surface marker of exosomes, can favor the budding of ILVs by interacting with a cluster of other tetraspanins and proteins 56, 57. Although several distinct exosome generation cellular pathways have been reported, the regulatory mechanisms within cells have not yet been elucidated.

The formation of ILVs follows the transport of MVEs toward the plasma membrane. Various intracellular trafficking molecules have been shown to participate in this process, including the cytoskeleton, molecular motors, and Rab GTPases. The final step is the fusion of MVEs with the plasma membrane for which the soluble N-ethylmaleimide-sensitive fusion attachment protein receptor (SNARE) complex is believed to be essential 59-63. The interaction between vesicle-membrane SNAREs (v-SNAREs) and target-membrane SNAREs (t-SNAREs) initiates the SNARE complex assembly, presumably allowing the fusion of MVEs with the plasma membrane and leading to exosome secretion.

#### MV generation

MV generation consists of two crucial steps: plasma membrane blebbing and scissoring. Plasma membrane rearrangement involving lipid and protein composition remodeling is the first essential step for membrane budding, and is believed to be a calcium (Ca^2+^)-dependent process 64. A group of Ca^2+^dependent enzymes, including flippases, floppases, and lipid scramblases, are involved in the rearrangement of membrane phospholipids 65. Mechanistically, phospholipid redistribution and maintenance can induce membrane lipid asymmetry and alter membranous curvature 66, 67. Besides lipid redistribution, unlocking the plasma membrane-cytoskeletal anchorage is necessary for membrane blebbing and vesiculation. In this respect, calpain, a Ca^2+^activated cysteine protease, can disrupt the attachment between the plasma membrane and cytoskeleton by cleaving several cytoskeletal components under the plasma membrane, such as actin and filamin 68, 69. However, our understanding of MV generation is limited and further mechanistic investigation is required.

In addition to the EV biogenesis machinery, recent studies suggest that several types of cell death, such as apoptosis, necroptosis, pyroptosis, and neutrophil extracellular trap formation (NETosis), are associated with EV generation, indicating the involvement of additional sophisticated mechanisms modulating EV biogenesis 70-76. EVs secreted by necroptotic cells mediate MLKL release, which can, in turn, serve as a self-control mechanism of necroptosis 77. These findings collectively indicate that EVs can function as a specialized intra- and inter-cellular messaging system, highlighting the importance of illustrating EV generation mechanisms.

### Sorting mechanism of EVs

Bioactive molecules, including proteins, DNAs, mRNAs, non-coding RNAs (ncRNAs), and metabolites, are encapsulated in EVs. Accumulating evidence suggests that cargoes are not randomly packaged into EVs or simply replicate the composition of their parental cells 78. Because of the significance of cargoes in signal communication, the mechanisms of cargo sorting of EVs are central to shed light on the physiological and pathological functions of EVs and their therapeutic implications. Although these mechanisms are far from being fully elucidated, recent advances provide exciting insights into this topic.

Recent studies have provided some clues on the sorting of proteins. First, ESCRT components and their related proteins can recruit exosomal cargoes through direct molecular interactions. For example, the ESCRT-I component TSG101 can recruit BAG6 into EVs, possibly playing a key role in directing EV proteins 79. In addition, the noncanonical ESCRT-dependent syntenin pathway also contributes to the sorting of specific exosomal cargoes, including LMP1 and KRS 80, 81. Second, common protein markers of EVs, particularly tetraspanins, have been suggested to account for sorting a great proportion of the exosomal proteins 82-85. High-throughput proteomic analysis of potential proteins interacting with tetraspanin-enriched microdomains revealed a significant overlap between the tetraspanin interactome and exosomal proteome, highlighting tetraspanins as important sorting machinery for protein inclusion into exosomes 82. Third, specific post-translational modifications (PTMs) seem to be emerging determinants for protein sorting in EVs, such as ubiquitylation 86-91, sumoylation 92, palmitoylation 93-96, farnesylation 97, phosphorylation 98-100, glycosylation 101-103, and lipidation 103. For instance, there was a 60% reduction of total protein levels in EVs derived from ubiquitin-like 3 (UBL3)-knockout mice, and UBL3 could function as a PTM factor by directly interacting with more than 1,200 proteins 86. Also, ESCRT components HRS, STAM, and TSG101 with their ubiquitin-binding domains might participate in ubiquitination 104. These observations emphasize the significance of ubiquitination, one of the most common PTMs, in sorting EV protein cargoes. Interestingly, ubiquitinated proteins have also been detected in the EVs secreted by insulin-secreting β cells, indicating a potential involvement in EV-associated islet cell dysfunction and T2D pathogenesis 105.

In addition to protein cargo, EVs carry a rich diverse RNA cargo, involved in many EV functions. The enrichment of distinct RNAs in EVs responsive to different cellular statuses relies on the sophisticated RNA sorting system. Recent studies suggest that the selective sorting of RNA in EVs is attributed mainly to RNA binding proteins (RBPs), accounting for about 25% of the protein content in EVs 106,107. RBPs usually recognize RNAs with specific “tags”, such as certain motifs, modifications, structures, or sequences, and sort them into EVs 107,108. For instance, hnRNPA2B1, a well-known regulator of RNA metabolism, can package specific miRNAs (miR-198, miR-30b-3p) and long noncoding RNAs (lncRNAs) (*AFAP1-AS1*, *LNMAT2*) into EVs by the direct interaction between its RNA-binding domains and the GGAG motif of the RNAs 109-113. Besides hnRNPA2B1, other members of the hnRNP family, including hnRNPA1, hnRNPC1, hnRNPH1, hnRNPK, hnRNPQ, and hnRNPU, have been implicated in RNA sorting and enrichment in EVs 114-120. Additionally, YBX1 (miR-223 in HEK293T cells), human antigen R (HuR) (miR-122 in human hepatic cells), and other RBPs have been reported to play a role in the selective miRNA enrichment in EVs 121-123.

The exploration of EV cargo sorting machinery is not restricted to EVs *per se*. An exciting correlation of autophagy with EV biogenesis and content loading has been recently reported, in which the LC3-conjugation machinery is proposed to govern the RBP capture and thus specify RNAs in secreted EVs 124-126, adding another layer of complexity to EV cargo sorting. In summary, EVs are emerging as an important mediator of intercellular communication. Elucidation of the mechanism underlying EV generation and content packaging has been an active area of research.

## EV biogenesis machinery in DM and diabetic complications

**Figure 3:**
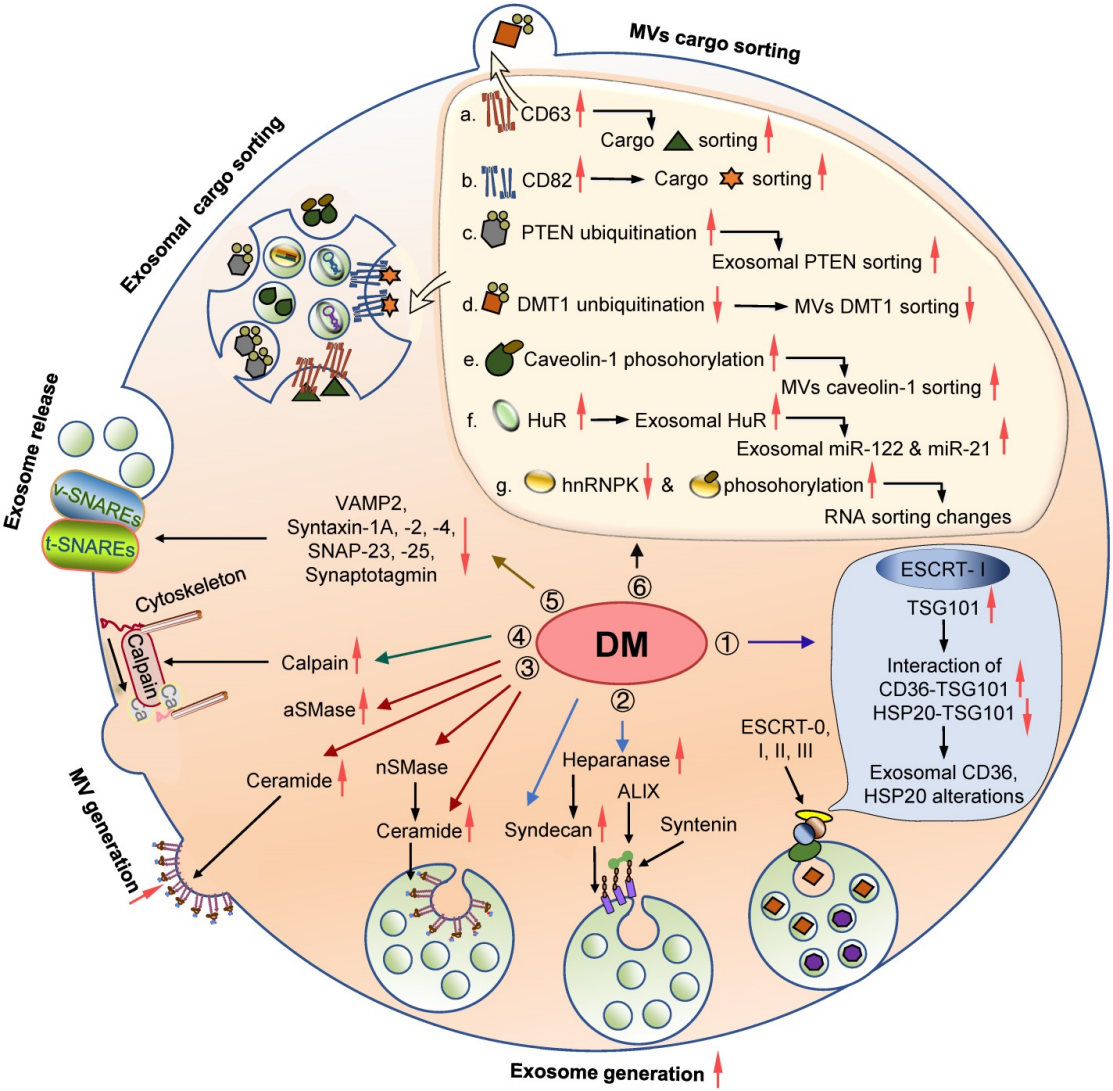
** Involvement of the EV biogenesis and cargo sorting machineries in DM and diabetic complications.** Diabetic conditions trigger the alteration in the expression and activity of the molecules involved in the process of EV biogenesis and cargo sorting. 1. Lipotoxicity induces TSG101 expression and influences its interaction with CD36 and HSP20, leading to their exosomal sorting dysregulation; 2. Elevated syndecans and heparinase in DM animals and patients can potentially activate of the syntenin-syndecan-ALIX pathway and promote exosomes biogenesis; 3. Elevated ceramide levels and nSMase/aSMase expression and activity may induce EV generation; 4. High glucose may impact the expression and activity of calpain 1 and 2, leading to elevated microvesicle generation; 5. Reduced SNARE components in diabetic conditions may influence exosomes release; 6. Altered expression of some regulators associated with EVs cargo sorting, as well as certain PTMs of specific proteins, may also affect EVs proteome and RNA profile under DM conditions. **Abbreviations:** aSMase: acid sphingomyelinase; DM: diabetes mellitus; ESCRT: endosomal sorting complex required for transport; EVs: extracellular vesicles; HuR: human antigen R; MV: microvesicle; nSMase: neutral sphingomyelinase; SNARE: soluble N-ethylmaleimide-sensitive fusion attachment protein receptor; t-SNAREs: target-membrane SNAREs; v-SNAREs: vesicle- membrane SNAREs.

EVs are crucial information transmitters between original and recipient cells, and their abnormalities contribute to the development of DM and diabetic complications. Exploring the mechanisms underlying EV biogenesis and cargo sorting is critical in developing novel therapies for various diseases. So far, the role of EV biogenesis and sorting machinery in diabetic pathology has not been systemically reviewed. Here we summarize current knowledge about the EV machinery involved in the pathogenic process of DM and its complications (Table 1 and Figure 3).

### EV generation

#### ESCRTs

ESCRT complexes participate in the generation of the majority of EVs. Multiple components of ESCRT complexes have been shown to play a role in various metabolic processes, especially glucose and lipid metabolism, indicating their potential involvement in DM and diabetic complications. Therefore, it is a reasonable assumption that EVs may partially mediate ESCRT functions in metabolism and metabolic diseases, albeit direct evidence is currently limited.

ESCRT complexes are involved in the transportation of lipid droplets and translocation of GLUT4 and glycogen synthase kinase 3β (GSK3β) in adipocytes, thereby mediating the regulation of neutral lipids micro-autophagy consumption, adipogenesis, and insulin-stimulated glucose uptake 213-215. Disruption of these cellular biological processes is involved in the pathogenesis of DM. Lipotoxicity, a common risk factor for IR and T2D, can induce TSG101 expression in adipocytes and thus promote the biogenesis of exosomes 216. Subsequently, TSG101 upregulation triggers the sorting of CD36 into EVs, which then are delivered into hepatocytes and evoke hepatic lipid accumulation 216. Furthermore, several factors interacting with ESCRT components may regulate EV formation, such as MLKL and HSP20 217-219. MLKL, a critical factor involved in plasma membrane disruption and necroptosis, is upregulated in multiple tissues, including the adipose, liver, muscle, kidney, and cardiomyocytes under diabetic conditions 220-224. MLKL can engage in the biogenesis of both exosomes and MVs by binding ESCRT proteins (TSG101, MVB128, VPS28, VPS37A, VPS25, CHMP3, CHMP4B, and CHMP2A) 218,219. Interestingly, MLKL can also regulate insulin sensitivity in diabetic mice independent of its proinflammatory and necroptotic roles 220. These observations indicate that non-necroptotic functions of MLKL might be mediated by its effect on EV formation.

In contrast to MLKL, HSP20 is downregulated in T1D and T2D and its reduction is considered a primary driver for DM-induced organ damage. HSP20 function, at least partially, is attributed to its regulatory activity on exosomes. Specific overexpression of HSP20 in cardiomyocytes can increase the generation/secretion of exosomes enriched in HSP20, p-AKT, survivin, and SOD1 through interacting with TSG101, thereby attenuating cardiac dysfunction, hypertrophy, and microvascular rarefaction under diabetic conditions 217,225. Besides the role of ESCRT components in metabolism and metabolic disease, it is anticipated that the crosstalk between ESCRTs and EVs may be involved in the pathogenesis of DM and its complications.

#### Ceramide and SMases

EVs are enriched in cholesterol and sphingolipids, such as sphingomyelin and hexosylceramide, and have a remarkable ceramide enrichment. Neutral sphingomylinase (nSMase) and acid SMase (aSMase) potentially mediate the budding of vesicles into MVEs and plasma membrane, respectively, and thus promote the generation of exosomes and MVs 55,226. Accumulating evidence suggests a role of the SMase-ceramide pathway in the pathogenesis of DM and its complications, although direct experimental data supporting EV contribution are lacking 127-141.

An elevated level of circulating ceramide is associated with the severity of IR in obesity 227. Specifically, membranous ceramide can influence the structural organization of plasma membrane and insulin receptor translocation, impairing insulin signaling 228, 229. In parallel, ceramide metabolism is over-represented in the plasma and markedly associated with the progression of T1D, consistent with its crucial role in immune regulation 230. Ceramide also serves as a critical lipotoxic mediator and drives the development of vascular dysfunction and damage 231-233. Similarly, abnormality and dysfunction of both aSMase and nSMase have been reported in DM and its complications (Table 1) 127-141. The pathogenic roles of these enzymes have generally been attributed to mediating sphingomyelin hydrolysis and ceramide in the AT, retina, liver, kidney, and other tissues. For example, aSMase and nSMase are increased in obese epididymal fat, along with altered levels of sphingomyelin, ceramide, and downstream ceramide metabolites in AT and plasma, promoting the expression of prothrombotic and proinflammatory genes and subsequently contributing to obesity-associated metabolic and cardiovascular diseases, such as atherosclerosis 127-129. Thus, inhibition of SMase-ceramide is considered an effective therapy for IR and DM by inhibiting inflammatory responses 131,132,234. However, the contribution of EVs in SMase-ceramide-mediated functions remains unknown and awaits future investigation.

#### Syndecan-syntenin pathway

The syndecan-syntenin-ALIX axis has been shown to regulate the formation of ILVs and exosomes 235. Syndecan, syntenin, and ALIX co-exist in a subset of exosomes. The PDZ domains of syntenin have a high affinity to syndecan, which recruits syntenin to membranes, while the N-terminal domain of syntenin directly interacts with ALIX. Heparanase, the only catalytic enzyme of syndecan, trims its heparan sulfate and significantly promotes exosome budding and generation 236. Syntenin can also recognize ligands with PDZ-binding motifs, which are specifically sorted into the exosomes. For example, syntenin directly binds the exposed PDZ-binding motif of KRS and targets it into exosomes, thereby contributing to caspase-8-triggered inflammation 80. These recent findings collectively suggest an important role of the syndecan-syntenin pathway in the biogenesis and function of exosomes.

Syndecan is a ubiquitous transmembrane protein and plays important physiological and pathological roles in development, differentiation, and human diseases, including DM and its complications (Table 1) 142-154. Generally, syndecan, particularly syndecan-1 and syndecan-4, are upregulated in diabetic humans and animals compared with euglycemic controls. Syndecan-1 is induced in the liver of obese Zucker fa/fa rats and potentially promotes lipid uptake, resulting in hepatic IR and dyslipidemia 142. Moreover, elevated syndecan-1 expression is associated with body mass index (BMI) and serum apoA1 in T2D, suggesting its involvement in vascular inflammation and injury 143-145. In T1D, syndecan-1 expression is positively correlated with microalbuminuria and inflammatory indicators, implying a role in DN pathogenesis 147, 148. Syndecan-4 is also increased in the heart and kidney of diabetic mice and rats and has a role in diabetic cardiomyopathy and DN 152-154.

The expression and activity of heparanase, a unique endoglycosidase known to degrade heparan sulfate chains, including those of syndecan-1, are increased under diabetic conditions 155-170. Notably, heparanase derived from insulitis leukocytes can degrade heparan sulfate of β cells and thus promote islet cell death in T1D. In mice, inhibition of heparanase can effectively delay the onset of T1D induced by STZ and NOD 155-158. Also, the level of heparanase in the circulation and urine is positively correlated with glucose and HbA1c 159-161 and is also closely associated with albuminuria in DM, indicating its crucial role in diabetic renal injury 162-167. Specifically, heparanase can potentially lead to the loss of heparan sulfate in the glomerular basement membrane, induce glomerular inflammation, and promote renal fibrosis in DN 150,162-167. Similarly, elevated heparanase has also been implicated in diabetic microangiopathies, such as diabetic retinopathy (DR) 150,168,169, and carotid artery atherosclerosis 170.

Together, these findings highlight the key roles of syndecan and heparanase in the pathogenesis of DM and its complications. Given the importance of the syndecans-syndecan-ALIX pathway in exosome biogenesis and cargo sorting, it is conceivable that exosomes could, at least partially, mediate syndecans and heparanase functions under diabetic conditions despite a lack of direct evidence.

#### Calpain

Calpains are a superfamily of Ca^2+^-dependent intracellular cysteine proteases and have a role in generating MVs *via* remodeling the cytoskeleton and facilitating the budding of the plasma membrane. Emerging data suggest that calpains, particularly calpain 1, 2, and 10, contribute to the genetic causes and biochemical defects of T2D, albeit a clear involvement of EVs in calpain-modulated T2D phenotypes remains elusive 171-191.

*CAPN10* encoding calpain 10 is the first positionally cloned gene for T2D 237-244. Its polymorphisms are closely associated with chronic diabetic vascular complications, such as DN, DR, diabetic neuropathy, and cardiovascular diseases 245. By utilizing multiple calpain inhibitors, recent studies have uncovered the function of calpains in IAPP-mediated cell dysfunction, insulin secretion in islet cells, insulin-stimulated glucose uptake, and glycogen synthesis in adipocytes and skeletal muscle cells 246,247. Notably, O-GlcNAcylation modification may facilitate the exosomal release of calpain 2 in hepatocytes under the high glucose (HG) condition 248. Exosomal calpain 2 can cleave the ectodomain of the insulin receptor and thus impair insulin action, providing a credible link between calpain 2, exosomes, and T2D etiology 248. Moreover, activation of calpain 1 and 2 contributes to accelerated atherothrombosis development in T2D by regulating different substrates in platelets and ECs 177-181. Since MVs loaded with elevated calpain 1 can be delivered to ECs and induce vascular inflammation 180,181, MVs might contribute to the phenotypes mentioned above.

#### SNARE proteins

The assembly of the SNARE complex mediates MVE fusion with the plasma membrane and allows exosome secretion into extracellular space. The role of SNAREs in glucose metabolism and T2D pathology has been extensively reported, although the involvement of EVs in the SNAREs-mediated effects remains unclear and awaits further investigation 192-198.

SNAREs fundamentally maintain glucose homeostasis *via* participating in insulin and glucagon-like peptide 1 (GLP-1) secretion and GLUT4-mediated glucose uptake 249-251. Many SNARE components, including VAMP2, syntaxin-1A, -2 and -4, SNAP-23 and -25, and synaptotagmin, are decreased in human and rodent T2D islets 192-195, and are associated with β cell hypertrophy and defective insulin secretion. Abnormal expression of SNARE proteins is implicated in IR in insulin-responsive tissues like the AT and muscle, probably due to impaired GLUT4 intracellular translocation 196, 197. In addition to dysregulated expression, abnormal location of SNAREs may have a role in systemic metabolism and T2D development. For example, abnormal sorting of VAMP2 into lipid droplets leads to inadequate trafficking of GLUT4 on the plasma membrane and IR in adipocytes 252. Additionally, VAMP2 is elevated in serum and possibly induces autoimmune response and consequent insulitis, suggesting it as a potential autoantigen of T1D 199.

### EV cargo sorting

#### Protein cargo sorting

Proteomic analysis has uncovered that diabetic condition alters the protein composition of EVs of different origins 253-257. Therefore, the cellular expression and function of EV protein sorting machinery in response to diabetic stimulations could be attributed to proteomic alterations of EVs in DM. In addition to ESCRTs mentioned above, tetraspanins CD63 and CD82 that participate in EV protein cargo sorting 83-85, have also been implicated in developing DM and its complications 200-203, 258.

Under glucolipotoxic conditions, CD63 mediates stress-induced nascent granule degradation of insulin in β cells, thereby mitigating insulin secretion and accelerating T2D 258. Moreover, AGEs can induce the expression of CD63, the marker of platelet activation, and the CD63^+^ platelet level is elevated in T2D patients with progression of carotid wall thickness 200, 201. CD63 is also upregulated in diabetic patients with DN and contributes to renal cell death by inhibiting the Wnt/β-catenin signaling pathway 202. CD82 is highly expressed in diabetic skin tissue and possibly associated with diabetic chronic inflammatory and hypoxic state 203, albeit its precise role and mechanism in diabetic dermopathy remain elusive. Taken together, altered expression and function of CD63 and CD82 under diabetic conditions may contribute to selective enrichment of cargoes in EVs and consequently induce changes in the EV proteome profile.

Additionally, several PTMs of proteins are required for their sorting in EVs, possibly involved in ubiquitination of PTEN and DMT1, phosphorylation of caveolin 1, and other diabetic pathological changes 259-265. Specifically, the concentration of polyubiquitinated PTEN, which plays an important role in regulating renal fibrosis, is increased in the serum and urine of DN patients 260. It has been reported that ubiquitination at lysine 13 of PTEN is required for the selective enrichment of PTEN in exosomes 90, which may partially mediate the pathological role of PTEN in DN. Similarly, the release of DMT1 from MVs is mediated by Nedd4-2 ubiquitin ligase, suggesting a role of ubiquitination in the cargo sorting of EV proteins in the gut explant 91. Moreover, *in vivo* and *in vitro* studies have found that HG leads to elevated DMT1 levels in intestinal epithelial cells partially by inhibiting DMT1 ubiquitination and promoting DMT1 membrane translocation, resulting in increased iron uptake and iron loading 259. Together, it is reasonable to hypothesize that the ubiquitinated DMT1 located at the plasma membrane is sorted into the budding vesicles and secreted into the extracellular environment. In contrast, the deubiquitinated DMT1 is trapped within cells, leading to elevated expression of DMT1 in the diabetic intestine. Also, phosphorylation of caveolin-1, a scaffolding protein involved in protein sorting of MVs 100, has been shown to be important in DN development 261-265. Hypoxia induces the phosphorylation of caveolin-1 that can directly interact with hnRNPA2B1, facilitating the sorting of hnRNPA2B1 and its-associated miRNAs into MVs 100. Under diabetic conditions, HG promotes caveolin-1 phosphorylation in podocytes and glomerular mesangial cells (GMCs), resulting in renal cell apoptosis, inflammation, EMT, and glomerular matrix accumulation 261-265. Moreover, circulating MVs derived from diabetic rats can be delivered into vascular ECs and lead to elevated caveolin-1 levels in recipient cells 266.

#### RNA cargo sorting

RNA cargo affects many EV functions in various diseases, including DM and diabetic complications. Diabetic conditions induce alterations of mRNAs, miRNAs, lncRNAs, and circular RNAs (circRNAs) in EVs 267-270, primarily due to the dysregulation of numerous RBPs. HuR, an extensively studied RBP, is involved in EV RNA sorting by directly recognizing and binding RNAs bearing AU-rich elements, such as miR-122 and miR-21 121. Both HuR and its associated miRNAs have been implicated in the diabetic heart, DN, and DR developing 204-210.

In the context of DM, target proteins post-transcriptionally modified by HuR have been shown to play a role in the pathogenesis of diabetic complications like DN, DR, and diabetic cardiomyopathy 204-210. For instance, HuR can post-transcriptionally modulate the expression of several regulators involved in renal injury, such as claudin-1, IL-17, NOD2, NLRP3, CTGF, TGF-β1, and Snail 204-207. Similarly, pyroptosis, inflammation, oxidative stress, and EMT have been mechanically involved in HuR-mediated DN development and progression. Moreover, it has recently been shown that HuR can be delivered into cardiomyocytes and thus elicit inflammatory and profibrogenic responses, highlighting its importance in the diabetic heart 209.

It has been reported that miR-122 and miR-21, two miRNAs sorted by HuR into EVs 121, have a role in the diabetic heart 271-273. MiR-21 is significantly decreased in cardiomyocytes of diabetic mice and contributes to diastolic cardiac dysfunction by directly targeting gelsolin and consequent oxidative stress. In contrast, circulating levels of miR-21 and miR-122 are increased in T2D patients with heart failure 272,273, probably resulting from increased EV secretion triggered by HuR upregulation. Thus, miR-21 and miR-122 may be selectively encapsulated in the EVs *via* HuR and secreted extracellularly, leading to an increase in their extracellular levels while causing a decrease in their intracellular levels, possibly mediating the pathogenic effect of HuR in diabetic cardiomyopathy. Similarly, these two miRNAs could play a role in DN and DR 274-277. For instance, it has been shown that miR-21 encapsulated in EVs exerts a pro-angiogenic effect on ECs and promotes DR development 278, 279. Further research is required to clarify whether the HuR function in the pathogenesis of diabetic complications depends on EV-miRNA sorting.

HnRNPK is another RBP involved in the RNA sorting of EV and its phosphorylation can be induced by glucolipotoxicity, a classic metabolic abnormality associated with T2D 211,212. Phosphorylated hnRNPK can significantly modulate the expression of oxidative and inflammatory genes in β cells 211. HnRNPK expression is decreased in the kidney of T1D mice and can potentially mediate RAS activation and hypertension in T1D 212. The altered expression and post-translational modification of hnRNPK might lead to different RNA selection in EVs, possibly contributing to hnRNPK function in DM.

Collectively, it is reasonable to conclude that EVs may mediate specific pathogenic roles of EV generation machinery in the initiation and progression of DM and diabetic complications. Thus, elucidating the association between EV biogenesis and diabetic pathogenesis represents an attractive direction for future investigation, which would pave the way for developing novel targeted therapeutics for DM and diabetic complications.

## Roles of EVs in DM

EVs are emerging as novel effectors of intercellular and interorgan communication and play active roles in multiple pathophysiological situations of metabolic modulation like metabolic homeostasis, maintenance, and disturbance. Of note, EV-induced phenotypic and molecular alterations in target cells are often associated with the composition and origin of these microstructures. In this section, we summarize the diverse EV functions in DM pathology and diabetic complications in the context of the cellular origin of EVs.

### Adipocytes

**Figure 4:**
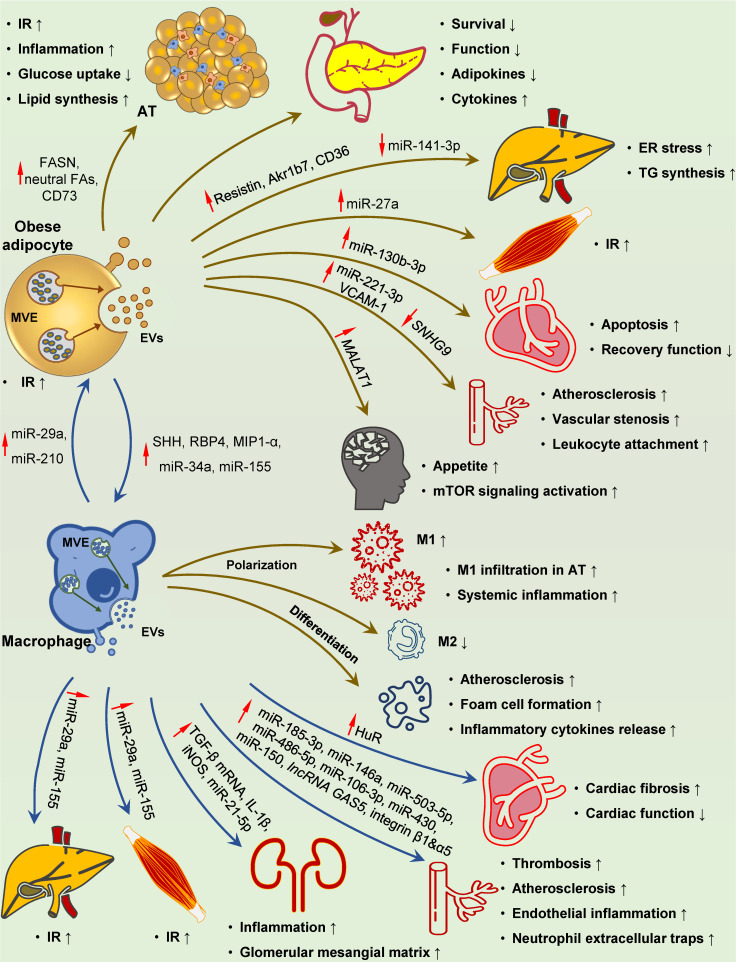
** Involvement of adipocyte- and macrophage-derived EVs in DM-related pathological changes.** Adipocyte-derived EVs play a distinct role at multiple processes in the development of DM-related pathology. These EVs with specific cargoes (FASN, neutral fatty acids, CD73, resistin, Akr1b7, CD36 and miR-27a) can circulate throughout the body and reach their destination for IR development and metabolic disturbance in the adipose, liver and skeletal muscle. Islet inflammation, damage and dysfunction can also be induced by adipocyte-derived EVs. Upon uptake by recipient cells, these EVs can deliver several pathogenic mediators to ECs, hypothalamus and heart (increased miR-221-3p and VCAM-1, reduced *SNHG9* to ECs, and increased *MALAT1* to hypothalamus), resulting in vascular injury, elevated appetite, and myocardial damage, respectively. SHH-, RBP4-, MIP1-α-, miR-34a- and miR-155-containing EVs taken up by macrophages can promote M1 polarization and foam cell differentiation, while inhibit M2 polarization, leading to localized adipose and systemic inflammation, and accelerated atherosclerosis. Reciprocally, inflamed macrophage-derived EVs carrying elevated miR-210 and miR-29a can be transferred to adipocytes, causing IR in the adipose tissues. EVs containing miR-29a originated from macrophages can also be delivered to the liver and skeletal muscle, leading to IR in target organs. Elevated HuR, integrin β1 and α5, IL-1β, iNOS, TGF-β mRNA, miR-21-5p, miR-185-3p, miR-146a, miR-503-5p, miR-486-5p, miR-106-3p, miR-430, miR-150, and *lncRNA GAS5* in these EVs ultimately result in cardiac fibrosis and dysfunction, atherosclerosis, renal inflammation, and glomerular mesangial matrix accumulation. **Abbreviations:** AT: adipose tissue; ECs: endothelial cells; ER: endoplasmic reticulum; EVs: extracellular vesicles; FAs: fatty acids; HuR: human antigen R; IR: insulin resistance; SHH: sonic hedgehog; TG: triglyceride.

AT is central in regulating systemic insulin sensitivity, hypertrophic adipocyte-induced elevated FFA release, inflammation, and adipokine alterations that are the drivers of the whole-body IR in T2D. Lipogenic stimulus and excess fat expansion promote EV generation in obese adipocytes, which, in turn, contribute to IR and islet cell dysfunction *via* paracrine effect and/or distant action (Figure 4) 280-285. These EVs induce lipid droplets deposit by directly delivering neutral fatty acids 286, and promote lipid synthesis by transmitting the key lipid synthesis enzyme FASN, lipogenic-related miRNAs and mRNAs, and CD73 281-284, 287. *In vitro* experiments showed that EVs can impair insulin response and glucose uptake in recipient adipocytes 280. Besides the paracrine effect on local adipocytes, EVs secreted by adipocytes can result in peripheral IR and metabolic disorder by functioning as adipokine carriers 288. Hypertrophic adipocyte-derived exosomes loaded with resistin, a canonical obesity-related adipokine, triggered hepatic ER stress and liver steatosis 289. Several studies have described functional lncRNA (*MALAT1*), miRNAs (miR-27a, miR-141-3p) and proteins (CD36, and Akr1b7) encapsulated in adipocyte-derived EVs as novel adipokines that exert metabolic modulatory effects on distant organs 216, 290-293. These newly discovered adipokines are sufficient to induce hepatic lipid accumulation and IR in the liver and skeletal muscle 216, 292, 293. In addition to peripheral tissues, EV-encapsulated *MALAT1* can be transported to pro-opiomelanocortin neurons, increasing appetite and body weight 291. Additionally, adipocyte-derived EVs have a modulatory effect on the survival and function of distant islets by delivering specific miRNAs 285.

Macrophage infiltration in AT is a hallmark of obesity and contributes to chronic inflammation and subsequent IR 294. Obese adipocyte-derived EVs have been demonstrated to play a role in recruiting and activating circulating monocytes and polarizing resident macrophages toward the proinflammatory phenotype 287, 295-300. Interestingly, based on the specific interactions between surface proteins of EVs and recipient cells, EVs are preferentially taken up by circulating monocytes *in vivo* and promote macrophage activation and IR 52, 295. In addition, obese adipocyte-derived EVs can shuttle bioactive molecules, such as miR-34a and miR-155, into recipient macrophages, and thus promote pro-inflammatory M1 polarization and inhibit anti-inflammatory M2 polarization 296-298.

EVs can also function as a mode of communication between adipocytes and vascular ECs, which may be dynamically influenced by metabolic status 301, and have a role in cardiovascular complications. EVs derived from obese AT often exert detrimental effects on vascular cells, including ECs, vascular smooth muscle cells (VSMCs), and cardiomyocytes 302-308. It has been shown that miR-221-3p, miR-130b-3p, *lncRNA SNHG9,* and VACM-1 within the EVs can result in endothelium inflammation, vascular stenosis, unstable atherosclerotic plaque formation, and impaired cardiac recovery 304-307.

### Macrophages

Inflammatory macrophages infiltrated in AT lead to low-grade tissue inflammation, which is the key cause of IR in T2D 309. EVs derived from obese AT macrophages (ATMs) can serve as systemic inflammation factors and impair insulin signaling in distal organs (Figure 4). Metabolic regulatory miRNAs, such as miR-29a, miR-155, and miR-210, can be carried by EVs and delivered into insulin-responsive cells and organs *via* paracrine or endocrine routes 310-313. These miRNAs robustly regulate insulin action on AT, liver, and skeletal muscle and cooperatively modulate systemic glucose homeostasis 310-313.

Macrophage-derived EVs also play a role in DM complications. It has been reported that HG and RAGEs induce EVs production in macrophages 314-318. Biomolecular cargoes within these EVs, such as IL-1β, iNOS, HuR, miR-21-5p, miR-486-5p, and TGF-β mRNA can be transferred to target cells and subsequently induce renal and cardiac injury and dysfunction 314-319. In particular, two miRNAs closely related to cardiac fibrosis and diastolic dysfunction, miR-122 and miR-1246 320, 321, have been shown to be specifically sorted into EVs by the RBP HuR 121, 322, raising the possibility that some pathogenic effects of HuR may be mediated by miRNAs enclosed in macrophage-derived exosomes. The oxidized low-density lipoprotein (oxLDL) is known to induce M1 polarization of macrophages and foam cell formation in the arterial wall, two crucial atherogenic events in DM. Interestingly, recent studies suggest an important role of miRNAs carried by activated macrophage-derived EVs in atherosclerosis 323-332. Thus, EVs can effectively transmit pathogenic miRNAs to target cells, including VSMCs, ECs, neutrophils, and macrophages, leading to vascular stenosis, dysfunction, and inflammation that promote atherosclerosis and thrombosis (Figure 4) 323-329. Besides miRNAs, functional factors, including *lncRNA GAS5*, integrin β1A, and α5, loaded in EVs, also participate in the progression of vascular injury and cardiovascular diseases 331,332.

Macrophage-derived EVs have been implicated in multiple immune response processes 333-335 and can present dead cell-associated auto-antigens to dendritic cells, and activate an autoimmune response 333,336. Exosomes derived from M1 macrophages can also act on T cells, amplifying Th1 response and aggravating neuritis in Guillain-Barré syndrome 337. Notably, macrophages infiltrated in islets are the main source of free radicals and pro-inflammatory cytokines, inducing β cell death in T1D 338,339; however, the potential contribution of EVs in this process awaits further exploration.

### Hepatocytes

Lipid stress under obese conditions leads to abnormal fat accumulation and inflammation in the liver in T2D, contributing to localized and systemic IR and inflammation. EVs derived from hepatocytes with overnutrition participate in this process *via* paracrine and endocrine actions. For instance, increased geranylgeranylation of Rab27a in hepatocytes promotes vesicle docking toward the plasma membrane and the subsequent EV release into circulation 340. Specifically, let-7e-5p, with the greatest increase in EVs under a high-fat diet (HFD), can be transferred to adipocytes and increase lipogenesis and adipose expansion through targeting Pgc1α 340. When taken up by the pancreas, EVs can promote islet cell proliferation and participate in the compensatory response in the early onset of T2D 341. In addition, these EVs are enriched in proinflammatory molecules, including S1P, TRAIL, integrin β1, ceramide, miR-122, and miR-192-5p, which can induce inflammatory cell infiltration and inflammation by attracting circulating monocytes and polarizing macrophages toward pro-inflammatory differentiation in the liver 342-348.

Moreover, EVs derived from lipid-stressed hepatocytes can mediate the crosstalk between the liver and cardiovascular system and contribute to related complications. EVs shed by steatotic hepatic cells contain elevated miR-1 and miR-122, which can induce expression of adhesion molecules and diminish mitochondrial activity in target ECs and cardiomyocytes, resulting in atherosclerosis aggravation and cardiac function impairment 349, 350.

### Islet cells

Insulin-releasing cells are considered the main effectors of autoimmune response, and their destruction is the main cause of T1D. In the past few years, EVs derived from islet cells under inflammatory stress have underscored their pathogenic function in autoimmune insulitis of T1D. Inflammatory cytokines induce islet autoantigen enclosure 351-354 and RNA profile alteration 270,355 in these EVs. Several known canonical diabetic antigens, for e.g., GAD65, IA-2, ZnT8, GLUT2, and proinsulin, as well as the newly identified Gag antigen, can be effectively delivered to antigen-presenting cells (APCs), leading to T cell activation and autoimmune response 351-354. In addition, these EVs can transfer bioactive RNAs and proinflammatory molecules, such as MCP1 and IL-27, to immune cells 355-359, and thus might account for the activation of recipient immune cells, such as dendritic cells, macrophages, B lymphocytes, and T lymphocytes. EVs derived from inflamed islet cells may also impose a pro-apoptotic effect on neighboring β cells by paracrine action and horizontal transmission of pathogenic miRNAs (e.g., miR-375-3p and miR-21-5p) associated with pancreas injuries 360.

Bioactive miRNAs loaded in pancreatic β cell-derived exosomes can function as endocrine factors, whose level changes influence glucose homeostasis and T2D development. HFD, the common risk factor for obesity and T2D, can affect specific miRNA levels in β cell-derived EVs, such as an increase in miR-29 and a decrease in miR-26a 361-363. These exosomal miRNAs can be transferred to peripheral tissues and impair insulin signaling in recipient cells, and also be transmitted to circulating monocytes and macrophages and induce chronic low-grade inflammation 361-363. MiR-26a is widely expressed in human tissues and involved in the pathogenesis of various human diseases, including DM and its associated disorders 364-369. Under T2D conditions, miR-26a expression is decreased in β cells, subsequently reducing circulating exosomal miR-26a, impairing insulin sensitivity and metabolic homeostasis in the liver and AT, thereby promoting the development of T2D 363. In contrast, exosomal miR-29s and miR-29a derived from islet cells are induced by FFAs stimulation and inflammation 361,362. These two exosomal miRNAs are delivered to the liver and inflammatory cells, resulting in hepatic IR and systemic metabolic dysregulation and inflammation 361,362. Moreover, islet cell-derived EVs seemingly contribute to pancreatic failure in T2D and thus promote disease progression. Mechanistically, EVs may potentially facilitate IAPP aggregation and amyloid formation in pancreatic cells, resulting in cell death 370. Additionally, pancreatic cell-derived EVs have a role in DM complications. HG stimulation significantly increases miR-15a levels in exosomes isolated from pancreatic β cells that can be readily absorbed by retinal cells and induce ROS production and apoptosis in recipient cells, leading to DR 371.

**Figure 5:**
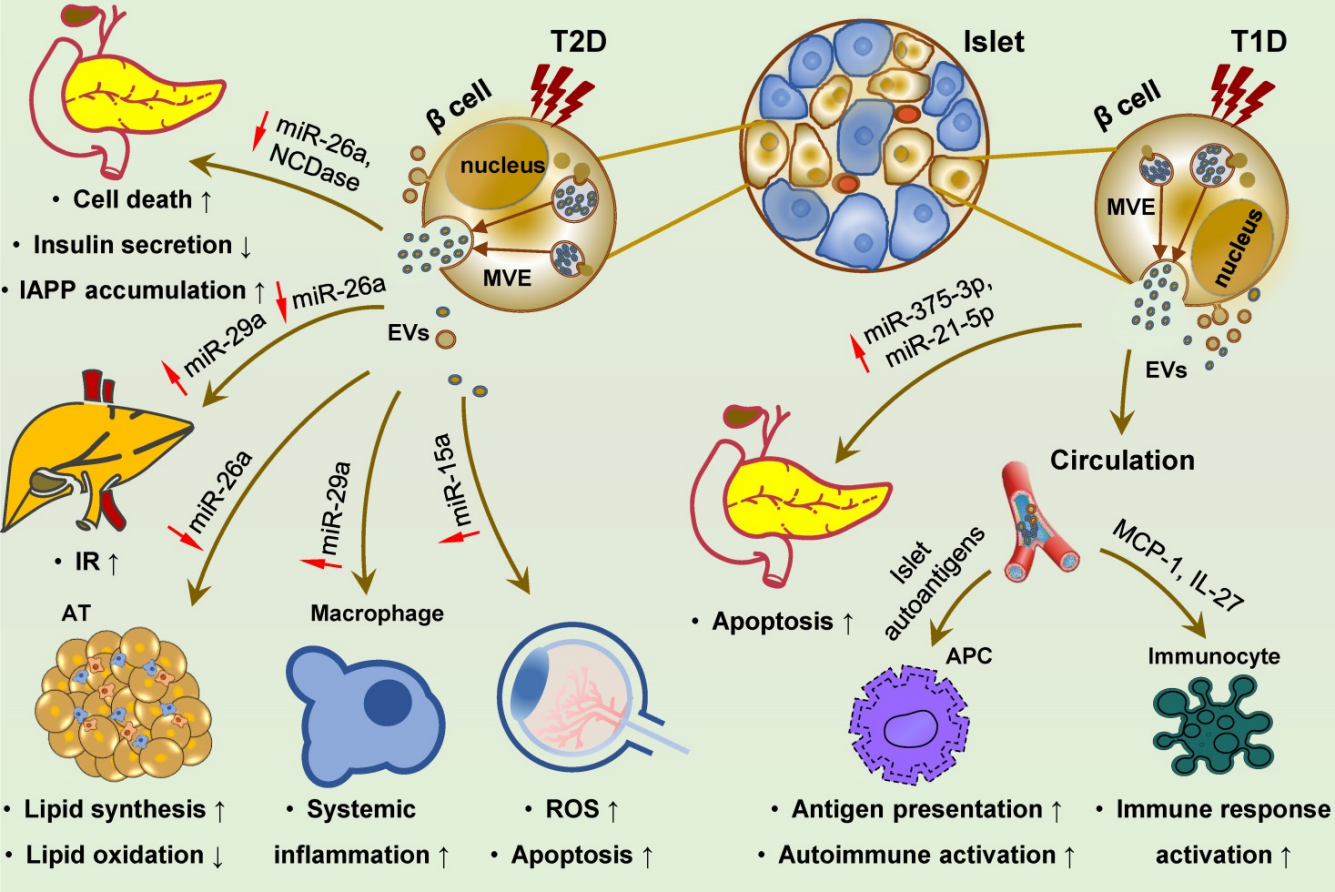
** Islet cell-derived EVs promote the development of T1D, T2D and diabetic retinopathy.** Islet cell-derived EVs carry various molecular effectors that can trigger multiple signaling cascades, and may regulate the development of T1D, T2D and diabetic complications. In T2D, reduced miR-26a and NCDase in these EVs can exert a paracrine effect on ambient islet cells, resulting in cell death, dysfunction and IAPP accumulation. Distant delivery of EVs derived from islets cells with reduced miR-26a and elevated miR-29s to the liver, adipose and macrophages can promote IR and lipid accumulation in the liver, and cell expansion and systemic inflammation in the adipose, ultimately leading to T2D development. EVs with increased miR-15a are also be transmitted to retina and cause oxidative stress and cell apoptosis, promoting the occurrence of diabetic retinopathy. In T1D, islets cell-derived EVs are encapsuled with islet autoantigens and facilitate autoantigen presentation and autoimmune activation, along with activating phagocytes and promoting cytokines and chemokines release. Inflammatory islet cell-derived EVs are loaded with increased miR-375-3p and miR-21-5p, exerting a pro-apoptotic effect on surrounding β cells via paracrine action. **Abbreviations:** APC: antigen presenting cell; AT: adipose tissue; EVs: extracellular vesicles; IAPP: islet amyloid polypeptide; IR: insulin resistance; ROS: reactive oxygen species; T1D: type 1 diabetes; T2D: type 2 diabetes.

To sum up, the pancreas is the target organ of diabetic injury and also serves as the pathogenic tissue releasing damaging EVs that can effectively mediate the crosstalk among the pancreas, distant organs, and immune system (Figure 5). Given that the pancreas is an active and potent endocrine and exocrine tissue and plays a central role in systemic metabolic homeostasis and multiple diseases, its EVs are expected to be involved in diverse physiological and pathological processes.

### ECs

**Figure 6:**
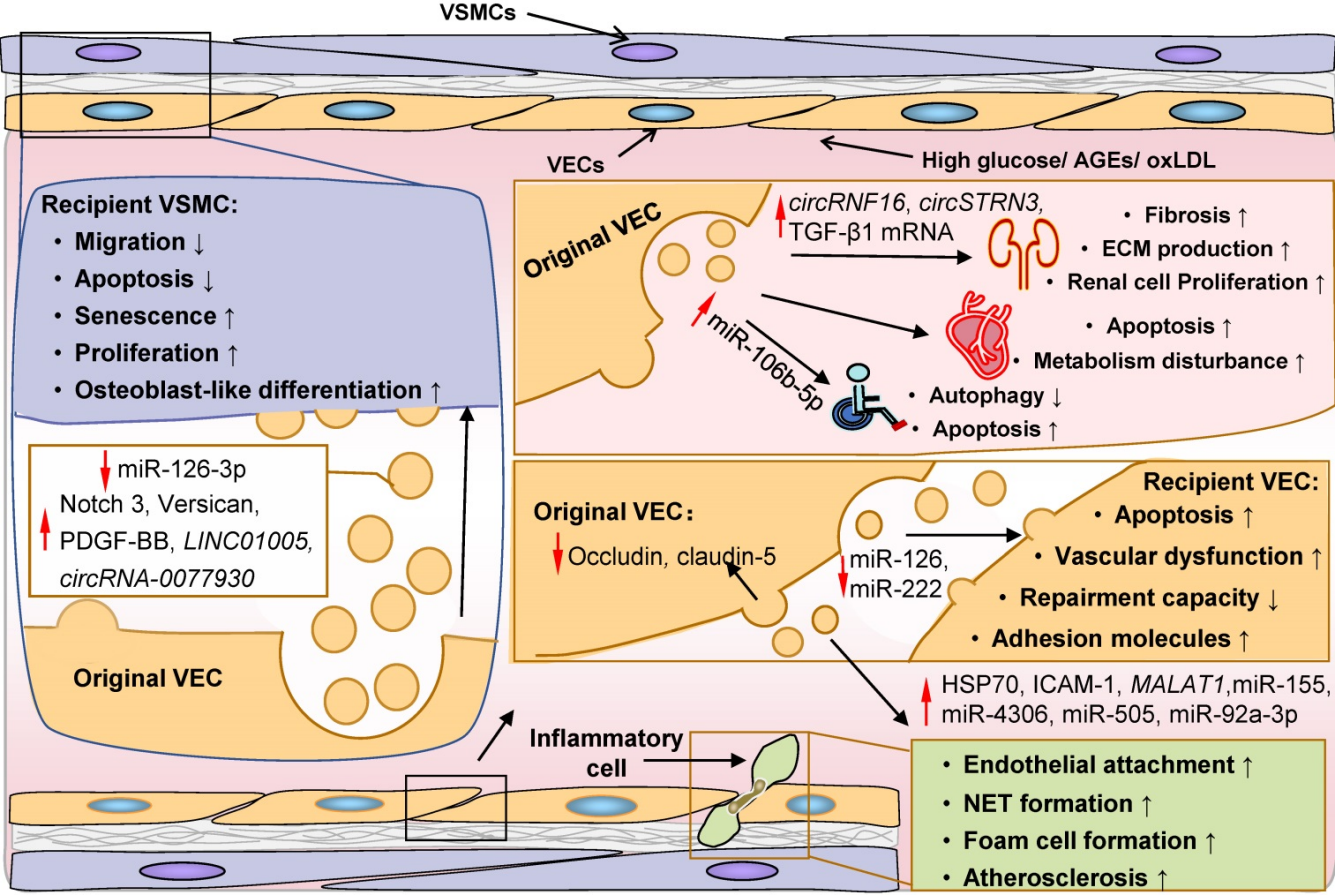
** Role of EC-derived EVs in the pathogenesis of diabetic complications.** EVs derived from ECs are critically involved in the occurrence and progression of diabetic complications, including endothelial damage and inflammation, vascular sclerosis, diabetic cardiomyopathy, diabetic nephropathy and diabetic foot, by transferring functional biomolecules. On the one hand, by secreting occludin and claudin-5 via EVs, original ECs lose tight junctions. On the other hand, EC-derived EVs can promote apoptosis, induce the expression of adhesion molecules, and impair repairment capacity of recipient ECs, resulting in endothelial injury and inflammatory cell attachment and infiltration in endothelium. The protective function of EC-derived EVs on endothelium (ECs and VSMCs) is potentially mediated by miR-126 and miR-222, which is decreased under diabetic conditions. Notch 3, versican, PDGF-BB, *LINC01005*, *circRNA-0077930* are delivered to VSMCs by EVs from ECs in a paracrine manner, resulting in apoptosis resistance and osteoblast-like differentiation in recipient VSMCs. EVs derived from ECs under oxLDL stress can transmit HSP70, ICAM-1, *MALAT1*, miR-155, miR-4306, miR-505 and miR-92a-3p into circulating system and local inflammatory cells including monocytes, macrophages and neutrophils, leading to endothelial inflammation and atherosclerosis. Glomerular EC-derived EVs are involved in the development of diabetic nephropathy via transferring TGF-β1 mRNA, *circRNF16* and *circSTRN3*, thereby promoting renal cell proliferation, fibrosis and ECM production. EVs derived from ECs can disturb energy metabolism and induce cardiomyocyte apoptosis, facilitating the development of diabetic cardiomyopathy. MiR-106-5p is increased in the EVs from ECs, and is subsequently transmitted into dermal fibroblasts and contributes to a refractory wound in diabetic foot. **Abbreviations:** AGEs: advanced glycation end products; ECs: endothelial cells; ECM: extracellular matrix; EVs: extracellular vesicles; NET formation: neutrophil extracellular trap formation; oxLDL: oxidated low-density lipoprotein; VEC: vascular endothelial cells; VSMC: vascular smooth muscular cell.

ECs are centrally involved in the microvascular pathology and complications in DM 372. Specifically, diabetic vascular complications are characterized by EC dysfunction and death, and endothelium inflammation. Accumulating evidence indicates that EC-derived EVs are involved in these processes *via* paracrine action (Figure 6). HG and AGEs have been shown to induce MV generation and alter EV cargo sorting in ECs 253, 373, 374. These MVs can promote apoptosis and dysfunction of recipient ECs 375-378. For example, reduced EV miR-126 and miR-222 are sufficient to decrease endothelium repair capacity, partially accounting for the loss of protective function of EC-derived EVs 376-378. Moreover, MVs are rich in membranous tight-junction proteins, occludin and claudin-5, resulting in a reduction of these molecules on the surface of parental ECs and impaired vessel walls 373. Additionally, these MVs can induce the expression of adhesion molecules in target ECs and facilitate inflammatory cells to attach and infiltrate into the endothelium 379, 380.

Capillary basement membrane thickening of the glomerular, retinal, cardiac, and cutaneous arterioles is the most common microvascular structural modification in DM, resulting in organ malperfusion and classic diabetic microangiopathy 372. In the diabetic setting, EVs derived from HG-treated ECs encapsulate elevated Notch3, versican, PDGF-BB, and *circRNA-0077930*, which can be taken up by surrounding VSMCs 381-386. Consequently, recipient VSMCs acquire an anti-apoptotic, osteoblast-like and senescent phenotype, leading to intimal hyperplasia and vascular calcification 381-386. Furthermore, ECs from different tissues can exert paracrine actions on ambient cells and promote the development of diabetic cardiomyopathy, DN, and diabetic foot. Exosomes derived from HG-treated ECs can suppress autophagy, increase apoptosis, and interfere with energy metabolism in target cardiomyocytes 387. Exosomes derived from diabetic glomerular ECs (GECs) transmit TGF-β1 mRNA to GMCs and podocytes then induce elevated proliferation and matrix production of GMCs and fibrosis of podocytes 388, 389. More recently, it has been shown that specific circRNAs in these exosomes, such as *circRNF169* and *circSTRN3*, may also contribute to the dysregulation of GMCs and mesentery proliferation in DN 269. Similarly, AGEs can boost miR-106b-5p in EVs derived from ECs that can be efficiently transported to recipient fibroblasts, leading to fibroblast autophagy and subsequent delayed wound healing 390.

Furthermore, generation and abnormal miRNAs sorting of EVs induced by oxLDL are also considered important atherogenic events in DM. Elevated EV miRNAs, including miR-155, miR-4306, miR-505, and miR-92a-3p, are delivered into macrophages, neutrophils and surrounding ECs, leading to endothelial inflammation, dysfunction, and damage, and promoting atherosclerosis 328, 391-393. Consequently, recipient inflammatory cells are aberrantly activated and exhibit a pro-inflammatory phenotype, while target ECs display decreased migration, proliferation, and angiogenic capacity 328,391-393. Besides, other bioactive molecules with an atherogenic role, such as *LINC01005*, *MALAT1*, HSP70, and ICAM-1, have also been detected in EVs and may play a role in DM pathogenesis 394-397.

### Other cells

Skeletal muscle is the major organ for glucose uptake, whose IR is one of the primary defects of T2D 398. During lipid-induced IR, exosomes derived from skeletal muscle cells are enriched in saturated fatty acid palmitate, which can be taken up by insulin-sensitive tissues, particularly the pancreas and liver, representing a new paradigm of inter-organ communication and metabolic homeostasis 399. MiR-16 encapsulated in these lipid toxic exosomes can promote the proliferation of target islet cells, acting as a compensatory IR mechanism during the onset of T2D 400. Nevertheless, after exercise training, skeletal muscle-derived EVs of healthy individuals carry specific protein and miRNA signatures and display liver tropism 401, 402. Bioactive miRNAs, including miR-133b, are transmitted to hepatic cells, inhibiting FoxO1 expression and leading to improved systemic metabolism 402. The target specificity is thought to be mediated by interactions between the proteins distributed on the surface of exosomes and recipient cells 401, 402.

Gut microbiota dysbiosis has a driving role in T2D by inducing abnormal intestinal metabolites and intestinal permeability dysfunction 403. Recent studies indicate that EVs derived from *Akkermansia muciniphila*, a beneficial bacterium preventing IR, contribute to the HFD-induced gut permeability elevation due to decreased intestinal tight junction function 404. In general, intestinal barrier disruption causes an increase in EVs derived from gut microbes in the circulation and whole body 405-408. The gut dysbiosis-related EVs appear to promote IR by transferring deleterious cargoes to recipient cells, such as HMGB1 and phosphatidylcholine 404-407.

In T1D, β cell death is primarily mediated by T cells, triggering diabetogenic insulitis 409. In addition to inflammatory cytokines that are traditionally viewed as inducers of islet mass loss, EVs loaded with pro-inflammatory miRNAs, such as miR-142-3p, miR-142-5p, and miR-155, have been shown to specifically target pancreatic β cells and function as a novel pathogenic factor mediating autoimmune attack of β cells in T1D 410.

In T2D, platelets are considered a mediator of cellular crosstalk and a driver of inflammation 411. EVs shed by platelets carrying soluble inflammatory cytokines have been recently implicated in these processes 412-414. In a diabetic setting, platelets can release more EVs containing increased CXCL7 and CXCL10 that could be targeted to ECs in the aorta, kidney, and retina, resulting in increased expression of adhesion molecules, ROS production, oxidative stress, and inflammation-induced endothelial injury, thereby promoting the development of DR, DN, and atherosclerosis 412-414.

EVs derived from the kidney also have a role in mediating intercellular crosstalk in diabetic conditions. On the one hand, HG and AGEs induce shedding of MVs from podocytes potentially *via* activation of NOX4/ROS and the Smad3 pathway 415, 416. These EVs mediate proximal tubular epithelial cell (PTECs) injury and apoptosis and proximal tubule fibrosis, partially due to transportation of miR-221 to target cells and subsequent regulation of Wnt/β-catenin signaling 415-419. On the other hand, HG-treated GMC-derived exosomes can be delivered to podocytes, which induce apoptosis and inhibit cell adhesion, leading to impairment of the last line of defense of the glomerular filtration barrier 420. These exosomes also potentially trigger an autocrine response in GMCs by delivering *circ-DLGAP4* and miR-15b-5p that induce fibrosis and apoptosis 421,422. Interestingly, HG seems to have a distinct effect on the generation of MVs and exosomes in PTECs. The MV release is increased under HG stimulation, which has a paracrine function on surrounding PTECs, promoting their fibrosis and impairing their adaptive responses combating hypoxia 423, 424. In contrast, exosome biogenesis is decreased by HG treatment, which then exhibits a pro-proliferative effect on target fibroblasts and promotes extracellular matrix production 425.

Exosomes derived from HG-treated retinal pigment epithelial cells can promote angiogenesis by directly delivering the pro-angiogenic factor VEGF into retinal ECs 426. Exosomes released by limbal stromal cells from non-diabetic individuals, but not from diabetic patients, can improve proliferation and migration of recipient limbal epithelial cells and maintain the integrity of cornea limbal epithelium 427. Additionally, diabetic condition disrupts the metabolism of Schwann cells (SCs), the most abundant cells in the peripheral nervous system, and results in their neurotrophic molecules production compromise, contributing to diabetic peripheral neuropathy 428. SC-derived exosomes act as an important neuronal support factor, nurturing peripheral axons and maintaining neuronal structure and function 429. Conversely, diabetic SC-derived exosomes likely function as carriers of pathogenic content, reducing the nerve conduction velocity and aggravating mechanical and thermal hypoesthesia in diabetic mice 430.

## Clinical applications of EVs in DM and diabetic complications

### EVs as a biomarker for DM

As described previously, EVs function as paracrine and endocrine factors and facilitate the crosstalk between metabolic organs and tissues. In addition, EVs have promising potential as biomarkers due to their good stability in body fluids and the ease of isolation and detection by fast-evolving technologies. Indeed, accumulating data have demonstrated the promise of EVs for clinical applications as biomarkers in DM. Several recent reviews, extensively summarizing EVs as potential biomarkers for the early detection of DM and diabetic complications, stratification of patients, and response monitoring of treatment from different perspectives, are highly recommended 431-433. Given the emerging role of EV RNAs in DM, here we briefly summarize the application of EV RNAs, including mRNAs and ncRNAs, as clinical biomarkers for the identification of diabetic patients and disease management (Table 2) 434-451.

For example, urinary exosomal miR-424 is robustly associated with islet autoimmunity and could efficiently discriminate patients with T1D with an area under the receiver operating characteristic (ROC) curve (AUC) of 0.803. However, serum miR-424 showed a relatively low diagnostic accuracy and sensitivity of 43% 452, suggesting urinary exosomal miR-424 as a more efficient biomarker for early detection of T1D. Another cohort study found that the combination of miR-10b and miR223-3p in serum MVs can effectively predict the occurrence of T2D in individuals with pre-diabetes with an AUC of 0.884 451. Importantly, this correlation has been further confirmed in the validation set with an AUC of 0.807 451. It has recently been pointed out that during the serum sampling process, apoptotic MVs with surface membrane phosphatidylserine could be consumed and new populations of MVs generated 453. The authors indicated that these possible major changes in serum MVs might raise controversy over the results 453. Compared to serum, the sampling process for plasma is simple with relatively stable contents. In this regard, it has been proposed that plasma might be a better source of MVs for biomarker investigation.

### Native EVs for DM therapy

EVs have been used as carriers of therapeutic substances and the administration of exogenous EVs has great promise in diabetic treatment. The therapeutic potential of EVs in treating DM and its complications in animal trials have been summarized and discussed in recent reviews 432,454. Here, we briefly discuss recent advances and the prospect of native EV-based therapeutics in DM and its complications.

**Figure 7:**
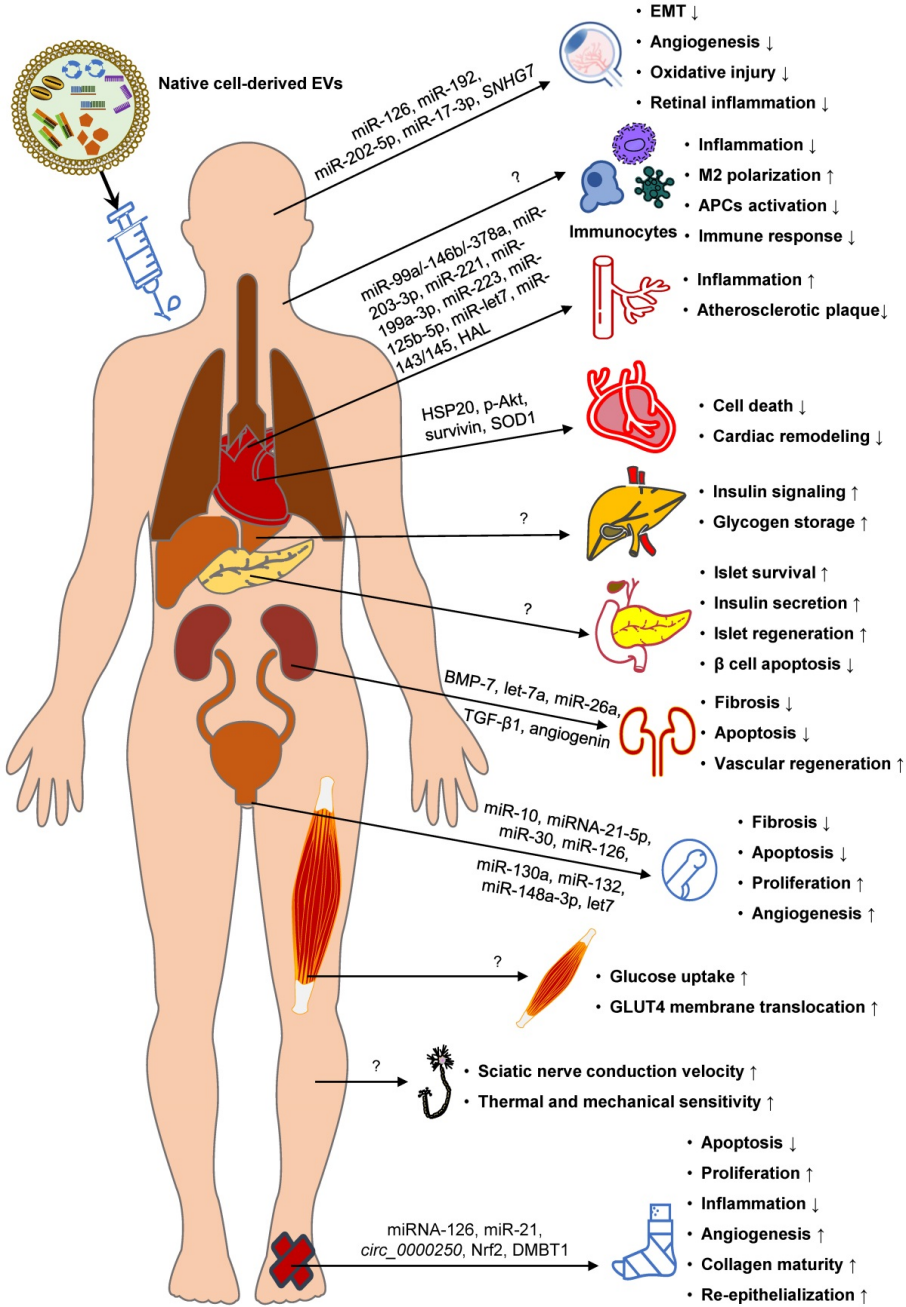
** Potential clinical applications of native cell-derived EVs in treating DM and its complications.** EVs of native cells (such as pancreatic pathfinder cells, adipocytes, stem cells, retinal pigment epithelial cells, keratinocytes, endothelial progenitor cells, amniotic epithelial cells, endothelial cells, fibrocytes, and macrophages) show potent promise as novel therapies for T1D (via inhibiting autoimmune response) and T2D (via promoting islet cell function and survival, and/or improving peripheral insulin sensitivity). These EVs also have the potential to treat diabetic complications including atherosclerosis, diabetic retinopathy, diabetic heart, diabetic nephropathy, diabetic erectile dysfunction, diabetic neuropathy, and diabetic foot ulcer. **Abbreviations:** APC: antigen presenting cells; DM: diabetes mellitus; EMT: epithelial-mesenchymal transition; ECM: extracellular matrix; EVs: extracellular vesicles; GLUT4: glucose transporter 4.

Anti-diabetic EVs have been isolated from various native cells, such as pancreatic pathfinder cells 455,456, adipocytes 457, and stem cells 458-462 (Figure 7). The preclinical data collected so far indicate that these EVs can improve peripheral insulin sensitivity and pancreatic islet function, alleviate inflammation, and/or attenuate obesity, regardless of their origin. The therapeutic roles of EVs in recipient cells have been ascribed to the delivery of bioactive proteins. For example, the anti-inflammatory and anti-apoptotic roles of exosomes have been attributed to active STAT3 and VEGF 459, 460. Depending on the source and content of EVs, they can trigger various therapeutic effects, such as inhibiting β cell apoptosis, restoring the phosphorylation of the insulin receptor substrate 1 and protein kinase B, increasing hepatic glycogen storage, polarizing M2 macrophages, and inhibiting the auto-immune response 458-462. Moreover, numerous examples of EV-mediated functional transfer of ncRNAs have been demonstrated for various diseases and the therapeutic applications of EV ncRNAs in treating DM offer a fertile field for study.

Another important clinical application of EVs from different origins is in treating diabetic complications, such as DN 463-466, DR 467-471, diabetic erectile dysfunction 472-475, diabetic foot 476-490, diabetic cardiomyopathy 217, atherosclerosis 491-500, and diabetic peripheral neuropathy 429 (Figure 7). Currently, the therapeutic roles of EVs in treating diabetic complications are mostly attributed to the delivery of ncRNAs, especially miRNAs. For example, the angiogenic role of EVs has been ascribed to miR-21, let-7, miR-10, miR-30, miR-148a-3p, miR-126, miR-130a, and miR-132 474-478, whereas their anti-fibrotic function has been attributed to let-7b and let-7c 474. Some of these miRNAs have been identified in previous studies as anti-diabetes therapies, such as miR-21 271,501,502, let-7 503, miR-126 504, and miR-132 505, 506, further highlighting their great potential in DM treatment. In addition to ncRNAs, some proteins like TGF-β1, angiogenin, BMP-7, Nrf2, and DMBT1within EVs can also elicit biological therapeutic effects 463, 482, 484.

However, important limitations in eliciting functional responses must be overcome for EVs to be used as an effective clinical therapeutic tool. Efforts have been made to address the challenges of harnessing the full potential of native EVs in the treatment of DM and diabetic complications. Because the EV composition is dependent on features of their donor cells, transfecting the original cells with exogenous compounds might modulate EVs and realize the goal of improving their bioactivity and augmenting their therapeutic efficacy. For example, overexpression of siFas and anti-miR-375 in human bone marrow mesenchymal stem cells can increase their levels in exosomes, effectively inhibiting Fas and miR-375 in recipient pancreatic islet cells and thus improve islet viability and function against inflammation 462. Similarly, overexpression of functional proangiogenic components, such as Nrf2 482, miR-221-3p 507, *mmu_circ_0000250* 480, and miR-126 476, in parental stem cells is accompanied by upregulation of these genes in the secreted exosomes, thereby improving the therapeutic effect against diabetic foot ulcer.

Furthermore, biomaterials, such as the thermosensitive and/or antibacterial hydrogel, have been developed to prolong the half-life of EVs and can serve as the controlled drug delivery system of EVs for treating chronic wounds 483, 508-510. Additionally, taking advantage of the high-yield EV-mimetic nanovesicles (EMNVs) as a novel drug delivery system, the nanocarriers loaded with *lncRNA-H19* have been applied to treat diabetic wounds 511. These EMNVs function effectively by restoring *lncRNA-H19* expression in dermal microvascular ECs and remarkably increase vascular formation 511 to treat DM and diabetic complications in the future.

## Conclusion and Perspective

EVs are major regulators of DM and diabetic complications and play an important role in IR, inflammation, and islet dysfunction. Significantly, EVs have shown promising efficacy in animal models to deliver bioactive proteins and RNAs and can be harnessed as effective therapies for DM and diabetic complications. Despite these tremendous advances, the basic and clinical research of EVs in DM and diabetic complications is still at an infant stage.

Although it is generally recognized that EVs communicate between cells and organs by delivering messages and exchanging information, many questions remain to be resolved. First, it is still challenging to categorize and characterize EV subclasses with high heterogeneity 512, mainly due to technological limitations in separating and analysing vesicles. Second, due to the complexity of EV contents, their functions, individually or collectively, are far from being fully elucidated. Many attempts have been made to address this issue, for e.g., by developing a single-vesicle array and imaging method to track EV uptake 513,514. Third, limited information is available about the molecular mechanisms underlying the target specificity of EVs with different origins so far. This process is believed to be largely mediated by membranous interactions between EVs originating from different cell types and target cells. Finally, it is important to monitor the fate of EVs after docking at recipient cells and determine the mechanisms underlying the usage of their cargoes.

From the clinical perspective, therapeutic applications of EVs have multiple challenges that need to be addressed. Biological detection of EVs requires adequate enrichment together with high sensitivity. Nanomaterials, such as magnetic nanoparticles, have been used to improve the sensitivity of EV detection 515, 516 by effectively increasing the interface between biological molecules and nanomaterials to facilitate the capture of target EVs, significantly raising the efficiency of EV isolation. Also, improving the specificity of EV separation required for the high specificity of biomarkers represents another challenge. Appropriate modifications of magnetic nanoparticles by attaching biological probes, such as antibodies targeting EV surface markers, can efficiently improve specificity 517,518.

However, there are several outstanding issues regarding the use of EVs as effective therapies for DM and diabetic complications, including scale-up of the production, shelf stability, prolonging the half-life of therapeutic EVs, toxicity, off-target effects, and the delivery specificity. Despite these problems, EV-based biomarker discovery and clinic application are feasible and promising with constantly developing technologies. Due to their unique biological characteristics, EVs still have a great potential for accurate early diagnosis of DM and overcoming diabetic complications.

## Figures and Tables

**Table 1:** Expressions and implications of EV biogenesis and sorting machinery under diabetic conditions.

Genes	Level/activity [Reference]	Sample: resource	Function
**aSMase**	↑ 127-129	AT: T2D patients with FLD, ob/ob mice	Promoting thrombosis and inflammation
	↑ 130,131	Serum: T2D patients, db/db mice	Promoting endothelial dysfunction
	↑ 132,133	Plasma, RECs, CD34^+^ CACs: T2D patients	Promoting inflammation and CACs migration
	↑ 134	RPECs: STZ rats	Impairing mitochondrial function
	↑ 135	Liver and brain: HFD mice	Promoting hepatic IR and neurodegeneration
	↑ 136	Kidney: GK rats	Promoting ER stress
**nSMase**	↑ 127-129	AT: T2D patients with FLD, ob/ob mice	Promoting thrombosis and inflammation
	↑ 135	Liver, brain: HFD mice	Promoting hepatic IR and neurodegeneration
	↑ 137	Skeletal muscle: Wistar fatty rats	Promoting IR in the muscle
	↑ 138	Vastus lateralis muscle: obese IGT patients	UD
	↑ 139,140	Islet β cells: Akita mice	Promoting β cell apoptosis
	↑ 141	Atrial appendage: obese T2D patients	UD
**Sdc1**	↑ 142	Liver: obese Zucker fa/fa rats	Promoting hepatic IR
	↑ 143-145	Neutrophils, serum: T2D patients	UD
	↓ 146	Serum, small intestine: STZ mice	Promoting epithelial barrier damage
	↑ 147,148	Plasma, serum: T1D DN patients	Promoting inflammation and microalbuminuria
	↑ 149,150	Vitreous fluid: PDR Patients	Promoting angiogenesis
	↓ 151	Skeletal muscle, heart: HFD ob/ob mice	UD
**Sdc4**	↑ 152,153	Heart, skeletal muscle: STZ rats	Promoting cardiac dysfunction
	↑ 154	Kidney: KK/Ta mice	UD
	↓ 151	Skeletal muscle, heart: HFD ob/ob mice	Promoting growth factor resistance
**HPSE**	↑ 155-158	Islet: NOD/Lt mice, T1D patients, STZ mice	Promoting β cell death
	↑ 159	Serum: obese patients with prediabetes	Promoting endothelial injury and inflammation
	↑ 160,161	Urine, plasma: T2D patients	UD
	↑ 162-167	Kidney: STZ mice and rats, DN patients	Promoting renal damage, protein excretion
	↑ 150,168,169	Vitreous fluid, serum, retina: PDR patients, STZ rats	Promoting inflammation, angiogenesis, and subendothelial barrier damage
	↑ 146	Serum, small intestine: STZ mice	Promoting epithelial barrier damage
	↑ 170	Carotid artery: DM patients, STZ rats	Promoting atherosclerosis
**Calpain**	↑ 171	Heart: STZ rats	Promoting apoptosis
	↑ 172-175	Heart: HFD, STZ, OVE26 mice	Promoting myocardial hypertrophy, and fibrosis
	↑ 176	Aortas: STZ and OVE26 mice	Promoting ROS and peroxynitrite production
	↑ 177,178	Platelet: T2D patients, STZ mice	Promoting platelets hyperaggregability
	↑ 179,180	Plasma: T2D patients	Promoting platelet activation and inflammation
	↑ 181	Platelet: T2D patients	Promoting MVs release and inflammation
	↑ 182	Dorsal root ganglion: STZ rats	Promoting oxidative stress and inflammation
	↑ 183	Penis: STZ mice	Promoting erectile dysfunction
	↑ 184	Lens epithelial cells: DR patients	UD
**Calpain-1**	↑ 185	Heart: STZ rats	Promoting oxidative stress and apoptosis
	↑ 186,187	Vascular mesentery: STZ and ZDF rats	Promoting endothelial inflammation
	↑ 188	Retina: STZ rats, HFD rats	Promoting retinal ganglion cell death
**Calpain-10**	↑ 189	Islet: T2D patients	Biomarker for islet dysfunction
	↑ 190	Kidney: STZ rats, HFD rats	Promoting apoptosis and renal dysfunction
	↓ 191	Kidney: STZ rats, ob/ob mice	Promoting apoptosis and renal dysfunction
**SNARE**	^a^ ↓ 192-195	Islet: T2D patients, GK rats, ZDF rats	Impairing insulin secretion
	^b^ ↓ 196	AT: STZ-NA rats	Promoting IR
	^c^ ↑ 197	Skeletal muscle: Zucker rats, STZ rats	Promoting IR
	^d^ ↓ 198	Hippocampus: STZ rats	UD
	^e^ ↑ 199	Serum: T1D patients	Promoting insulitis as autoantigen
**CD63**	↑ 200,201	Platelets: T2D patients	UD
	↑ 202	Kidney: DN patients	Promoting renal cell apoptosis
**CD82**	↑ 203	Skin: DM patients	Promoting chronic inflammation
**HuR**	↑ 204-207	Kidney: DN patients, db/db mice, STZ rats	Promoting pyroptosis, inflammation, and EMT
	↑ 208	Retina: STZ rats	Promoting angiogenesis
	↑ 209	BMMØ, heart: db/db mice	Promoting cardiac fibrosis and dysfunction
	↑ 210	Heart: diabetic cardiomyopathy patients	Promoting pyroptosis and inflammation
**hnRNPK**	PTM^f^ ↑ 211	Islet: db/db mice	Promoting oxidative stress and apoptosis
	↓ 212	Kidney: Akita mice	Promoting RAS activation and hypertension

AT: adipose tissue; BMMØ: bone marrow‐derived macrophage; CACs: circulating angiogenic cells; DN: diabetic nephropathy; FLD: fatty liver disease; GK rats: Goto-Kakizaki rats; HFD: high-fat diet; IGT: impaired glucose tolerance; HPSE: heparanase; NOD/Lt mice: nonobese diabetic mice harboring a hybrid rat insulin-promoter/SV40 large T-antigen gene spontaneously develop β-cell adenomas; OVE26: FVB(Cg)-Tg(Ins2-CALM)26OveTg(Cryaa-Tag)1Ove/PneJ transgenic mice; PDR: proliferative diabetic retinopathy; PTM: posttranslational modification; RAS: renin-angiotensin system; Sdc1: syndecan 1; Sdc4: syndecan 4; SNARE: soluble N-ethylmaleimide-sensitive fusion attachment protein receptor; STZ+NA: streptozotocin+ nicotinamide; UD: undetermined; ZDF: Zucker fat diabetic. ^a^: synaptotagmin, VAMP-2, syntaxin-1A and -2 and SNAP-25; ^b^: SNAP23, syntaxin-4 and VAMP-2; ^c^: VAMP-2, syntaxin-4; ^d^: syntaxin-1; ^e^: VAMP2; ^f^: phosphorylation.

**Table 2:** Diagnostic index of EV RNAs in DM and diabetic complications.

RNAs	Types [Reference]	Source	Number (ND/DM)	AUC	SEN (%)	SPE (%)	95% CI
**let-7c-5p**	T2DN 444	Urine	15/28	0.818	96	53.4	0.718-0.919
**miR-21-5p**	T2D 442	Plasma	60/57	0.859	-	-	-
	T2D-C 442	Plasma	57/101	0.744	-	-	-
	T2DN 438	Urine	15/14	0.830	-	-	0.673-0.986
**miR-23a**	T2D 434	Plasma	36/42	0.828	-	-	0.735-0.920
**miR-29c-5p**	T2DN 444	Urine	15/28	0.774	-	-	-
**miR-30a-5p**	T2D-ESRD 436	Urine	80/40	0.912	-	-	-
**miR-30a**	T2DN 443	Urine	56/110	0.897	76.4	90.9	0.858-0.936
**miR-34a**	Dyslipidemia 439	Serum	78/42	0.730	-	-	0.630-0.830
	T2DN 441	Urine	44/136	0.917	93.3	86.7	0.874-0.96
**miR-133b**	T2DN 443	Urine	56/110	0.867	86.4	72.7	0.820-0.914
**miR-146a-5p**	T2D 442	Plasma	60/57	0.911	-	-	-
	T2D-C 442	Plasma	57/101	0.673	-	-	-
**miR-156**	T2DN 441	Urine	44/136	0.883	97.8	82.2	0.824-0.942
**miR-156-5p**	T2DN 444	Urine	15/28	0.818	-	-	-
**miR-192**	T2D 434	Plasma	36/42	0.717	-	-	0.607-0.828
	MIC 440	Urine	30/30	0.802	-	-	0.696-0.907
**miR-194**	MIC 440	Urine	30/30	0.703	-	-	0.581-0.826
**miR-215**	MIC 440	Urine	30/30	0.757	-	-	0.545-0.869
**miR-218**	T1D 437	Urine	30/30	0.817	-	-	-
**miR-342**	T2DN 443	Urine	56/110	0.910	81.8	80.9	0.873-0.948
**miR-424**	T1D 437	Urine	30/30	0.803	-	-	-
**miR-636**	T2DN 441	Urine	44/136	0.984	97.8	93.3	0.971-0.997
**miR-4534**	DN 435	Urine	14/14	0.786	85.7	78.6	0.607-0.965
**miR-10b and miR223-3p**	T2D 451	Serum	8/9	0.884	-	-	-
***circ_0000907***	DFU 445	Serum	20/20	0.878	80	80.85	-
***circ_0057362***	DFU 445	Serum	20/20	0.848	86.005	70.22	-
**Ace**	Overt DN 448	Plasma	100/37	0.75	73	72	0.66-0.83
	Incipient DN 448	Plasma	37/66	0.62	65.2	61	0.54-0.71
**Aebp1**	T2DN 446	Plasma	15/15	0.742	53.3	86.7	-
**Ccl21**	T2DN 447	Urine	15/28	0.888	-	-	0.737-0.997
**Umod**	T2DN 450	Urine	15/88	0.90	93	73	-
**Wt1**	Incipient DN 448	Plasma	37/66	0.63	50	74	0.55-0.72
	Overt DN 448	Plasma	100/37	0.83	67.6	93	0.74-0.92
	DN 449	Urine	10/10	0.705	-	-	-

AUC: area under the ROC curve; CI: confidence interval; DFU: diabetic foot ulcer; DM: diabetes mellitus; DN: diabetic nephropathy; ESRD: end-stage renal disease; MIC: microalbuminuria; ND: non diabetes; SEN: sensitivity; SPE: specificity; T2DN: T2D with DN; T2D-C: T2D with complications.

## Abbreviation

aSMase: acid sphingomyelinase
AGEs: advanced glycation end products
APCs: antigen-presenting cells
AT: adipose tissue
AUC: area under the receiver operating characteristic curve
BMI: body mass index
circRNAs: circular RNAs
CNS: central nervous system
DM: diabetes mellitus
DN: diabetic nephropathy
DR: diabetic retinopathy
ECs: endothelial cells
EMNVs: EV-mimetic nanovesicles
ER: endoplasmic reticulum
ESCRT: endosomal sorting complex required for transport
EVs: extracellular vesicles
FFAs: free fatty acids
GECs: glomerular ECs
GLP-1: glucagon-like peptide 1
GLUT: glucose transporter
GMCs: glomerular mesangial cells
GSK3β: glycogen synthase kinase 3β
HG: high glucose
HFD: high-fat diet
HuR: human antigen R
IAPP: islet amyloid polypeptide
ILVs: intraluminal vesicles
IR: insulin resistance
lncRNAs: long noncoding RNAs
MVs: microvesicles
MVEs: multivesicular endosomes
ncRNAs: non-coding RNAs
NETosis: neutrophil extracellular traps formation
nSMase: neutral sphingomyelinase
oxLDL: oxidated low-density lipoprotein
PTECs: proximal tubular epithelial cells
PTMs: posttranslational modifications
RAGEs: receptors for AGE
RBPs: RNA binding proteins
ROC: receiver operating characteristic
ROS: reactive oxygen species
SCs: Schwann cells
SNARE: soluble N-ethylmaleimide-sensitive fusion attachment protein receptor
t-SNAREs: target-membrane SNAREs
T1D: type 1 diabetes
T2D: type 2 diabetes
UBL3: ubiquitin-like 3
v-SNAREs: vesicle-membrane SNAREs
VSMCs: vascular smooth muscle cells
